# Independent Effects of Blood Pressure on Intraocular Pressure and Retinal Ganglion Cell Degeneration: A Mendelian randomization study

**DOI:** 10.1167/iovs.65.8.35

**Published:** 2024-07-01

**Authors:** Skanda Rajasundaram, Ayellet V. Segrè, Dipender Gill, Benjamin Woolf, Seyedeh M Zekavat, Stephen Burgess, Anthony P. Khawaja, Nazlee Zebardast, Janey L. Wiggs

**Affiliations:** 1 Faculty of Medicine, Imperial College London, London, UK; 2 Department of Ophthalmology, Massachusetts Eye and Ear, Harvard Medical School, Boston, Massachusetts, USA; 3 Ocular Genomics Institute, Massachusetts Eye and Ear, Boston, Massachusetts; 4 Broad Institute of MIT and Harvard, Cambridge, Massachusetts, USA; 5 Department of Epidemiology and Biostatistics, School of Public Health, Imperial College London, London, UK; 6 School of Psychological Science, University of Bristol, Bristol, UK; 7 MRC Integrative Epidemiology Unit, University of Bristol, Bristol, UK; 8 MRC Biostatistics Unit, University of Cambridge, Cambridge, UK; 9 Yale University School of Medicine, New Haven, Connecticut, USA; 10 NIHR Biomedical Research Centre, Moorfields Eye Hospital NHS Foundation Trust and UCL Institute of Ophthalmology, London, UK

**Keywords:** intraocular pressure, blood pressure, primary open-angle glaucoma, retinal ganglion cell degeneration, Mendelian randomization

## Abstract

**Purpose:**

To investigate the causal effect of elevated blood pressure on primary open-angle glaucoma (POAG) and POAG endophenotypes.

**Methods:**

Two-sample Mendelian randomization (MR) was performed to investigate the causal effect of elevated systolic blood pressure (SBP) (*N*=757,601) and diastolic blood pressure (DBP) (*N*=757,601) on intraocular pressure (IOP) (*N*=139,555), macular retinal nerve fiber layer (mRNFL) thickness (*N*=33,129), ganglion cell complex (GCC) thickness (*N*=33,129), vertical cup-to-disc ratio (VCDR) (*N*=111,724), and POAG liability (*N*_*cases*_=16,677, *N*_*controls*_=199,580). The primary analysis was conducted using the inverse-variance weighted approach. Sensitivity analyses were performed to investigate robustness to horizontal pleiotropy, winner’s curse, and collider bias. Multivariable MR was performed to investigate whether any effect of blood pressure on retinal ganglion cell degeneration was mediated through increased IOP.

**Results:**

Increased genetically predicted SBP and DBP associated with an increase in IOP (0.17mmHg [95% CI=0.11 to 0.24] per 10mmHg higher SBP, *P*=5.18x10^-7^, and 0.17mmHg [95% CI=0.05 to 0.28mmHg] per 10mmHg higher DBP, *P*=0.004). Increased genetically predicted SBP associated with a thinner GCC (0.04µm [95% CI=-0.07 to -0.01µm], *P*=0.018) and a thinner mRNFL (0.04µm [95% CI=-0.07 to -0.01µm], *P*=0.004), an effect that arises independently of IOP according to our mediation analysis. Neither SBP nor DBP associated with VCDR or POAG liability.

**Conclusions:**

These findings support a causal effect of elevated blood pressure on retinal ganglion cell degeneration that does not require intermediary changes in IOP. Targeted blood pressure control may help preserve vision by lowering IOP and, independently, by preventing retinal ganglion cell degeneration, including in individuals with a normal IOP.

## Introduction

Primary open-angle glaucoma is a degenerative optic neuropathy characterised by the loss of retinal ganglion cells ^[1]^. IOP is an established causal risk factor for POAG and to date, lowering IOP remains the only proven way of slowing the progression of vision loss in POAG ^[1]^. Of the different inner macular retinal segments delineated by spectral-domain optical coherence tomography (SD-OCT) imaging, ganglion cell complex (GCC) and macular retinal nerve fiber layer (mRNFL) thickness measurements are two of the most sensitive and accurate biomarkers of early glaucoma ^[2] [3] [4]^. The GCC comprises the three innermost retinal layers: the RNFL, ganglion cell layer, and inner plexiform layer (with the latter two layers collectively termed the ganglion cell-inner plexiform layer (GCIPL)). GCC thickness is valuable in the early diagnosis of POAG ^[5]^ and in monitoring its progression ^[6]^. mRNFL thickness has also been shown to reliably predict glaucomatous visual field defects ^[7] [8]^. As direct measures of retinal ganglion cell loss, the pathological hallmark of POAG, these parameters may also help elucidate IOP-independent mechanisms driving the development of POAG. The vertical cup-to-disc ratio is another quantitative clinical biomarker that has consistently and specifically been shown to predict the onset and progression of POAG ^[9] [10] [11]^.

Various putative risk factors are associated with primary open-angle glaucoma and POAG endophenotypes however, many of these are either non-modifiable, e.g., age and ethnicity, or the extent to which they are truly causal rather than simply correlative is uncertain. Identifying those modifiable risk factors that exert a causal effect on these traits could help clinicians counsel patients at high risk of POAG on relevant lifestyle changes or preventative treatments. Elevated systemic blood pressure, which frequently coexists with POAG, is one such modifiable putative risk factor ^[12]^. Numerous observational studies report a positive association between blood pressure and IOP ^[13] [14] [15]^, and other studies have sought to investigate the relationship between blood pressure, inner macular thinning ^[16] [17] [18] [19].^ and VCDR ^[20] [21]^. However, the ability of conventional observational studies to infer causation is inherently undermined by unmeasured confounding and so the causal effect of blood pressure in the setting of POAG remains uncertain. Mendelian randomization (MR) exploits the random allocation of genetic variants at conception to infer the causal effect of an exposure on an outcome, robust to the influence of environmental confounding ^[22]^. MR is also robust to bias due to classical measurement error in continuous exposures, which often undermines traditional observational studies ^[23]^. However, MR considers associations using population-level genetic association data and so results are not necessarily translatable into causal effects for any given individual. Nevertheless, conditional on certain assumptions, MR can provide valuable genetic support for the presence or absence of a causal effect of long-term elevated blood pressure on POAG endophenotypes and POAG liability. Indeed, previous studies have leveraged MR to provide aetiological and therapeutic insight in the setting of POAG ^[24] [25] [26]^. We therefore used two-sample MR to investigate the potential causal effect of systemic blood pressure on these different traits. First, we used univariable MR to investigate the causal effect of systolic and diastolic blood pressure on IOP, mRNFL thickness, GCC thickness, VCDR, and liability to POAG. Given that associations between IOP and inner macular thinning have previously been reported ^[27]^, Multivariable Mendelian randomization (MVMR) mediation analysis was then conducted to investigate whether any identifiable effects of blood pressure on mRNFL or GCC thickness were mediated through IOP or arose independently of IOP.

## Methods

### Data Sources

**Figure 1:**
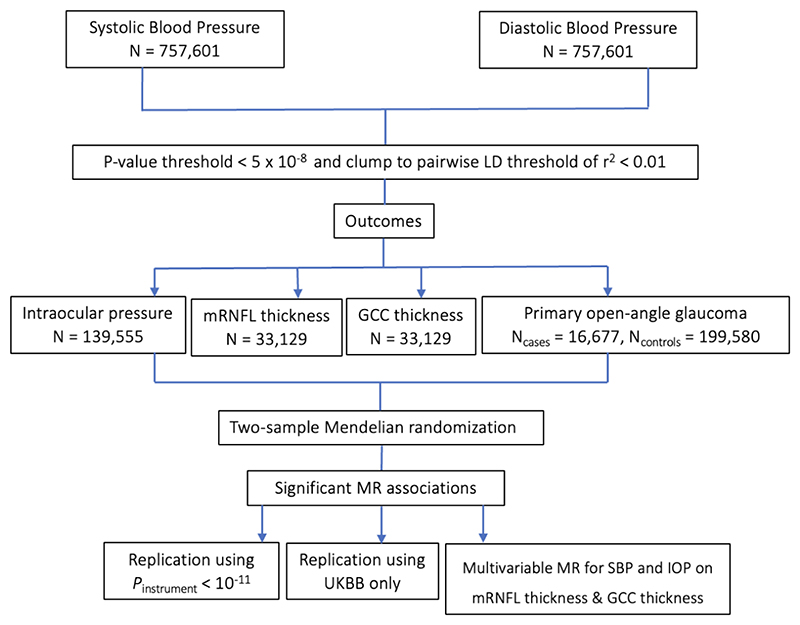
Study design. UKBB = UK Biobank. mRNFL = macular retinal nerve fiber layer. GCC = ganglion cell complex. MR = Mendelian randomization. Primary analysis used Evangelou *et al.’*s 2018 GWAS combining ICBP and UKBB data on SBP and DBP. Genetic instruments were selected after applying *P*-value and LD clumping thresholds. Two-sample MR was performed. Additional sensitivity analyses were performed, using a more stringent *P*-value threshold of *P* < 10^-11^ and using only UKBB data for SBP and DBP.

A flowchart illustrating the study design is shown in Figure 1 and a summary of the data sources are provided in Table S1. In the primary analysis, genetic association data for SBP and DBP were derived from Evangelou *et al*.’s GWAS meta-analysis of UKBB and ICBP (*N*=757,601) as these are the largest SPB and SBP GWASs conducted to date ^[28]^. These GWASs were adjusted for age, age^2^, sex, and BMI, and were corrected for antihypertensive medication use by adding 15mmHg. For IOP, data were obtained from the largest published IOP GWAS meta-analysis (*N*=139,555), combining data from the UK biobank (UKBB), EPIC-Norfolk and the International Glaucoma Genetics Consortium (IGGC), with adjustments made for age, sex, and the first five principal components ^[29]^. For mRNFL and GCC thickness, data were obtained from Zekavat *et al*.’s GWAS of spectral-domain OCT scans of the macula for 33,129 individuals in UKBB with adjustments made for age, age^2^, sex, smoking, spherical equivalent, the first ten principal components of genetic ancestry, and genotyping array ^[30]^. For VCDR, data were obtained from Han *et al*.’s GWAS of AI-derived VCDR adjusted for vertical optic disc diameter based on 282,100 images (*N*=111,724 individuals) from the UKBB, Canadian Longitudinal Study on Aging, and IGCC ^[31]^. Adjustments were made for age, sex, and the first ten principal components. This is the largest VCDR GWAS performed to date and, given the wide physiological variability in optic disc diameter, adjusting for optic disc size may increase the clinical utility of VCDR in diagnosing POAG ^[32]^. For POAG, data were obtained from the largest published POAG European ancestry GWAS meta-analysis (*N*_*cases*_=16,677, *N*_*controls*_=199,580) with adjustments made for age, sex, and study-specific principal components^[33]^.

All exposure and outcome GWASs included data from the UKBB, a large prospective cohort study of approximately 500,000 participants across the UK. On recruitment, participants had numerous biological and clinical variables measured, were genotyped with rigorous quality control checks, and consented to ongoing linkage of their medical records ^[34]^. Given the small amount of phenotypic variation typically explained by genetic instruments in MR, the UKBB therefore provides a well-powered resource of genotype-phenotype association data for use in MR analyses. Data for all exposures and outcomes were derived from European ancestry individuals. Informed consent for all participants was obtained in the original studies, which were granted relevant ethical approval. The study was reported in line with the ‘strengthening the reporting of observational studies in epidemiology using mendelian randomization’ (STROBE-MR) guidelines (Table S15) ^[35]^.

### Genetic Instruments

To proxy blood pressure, variants associated at *P* < 5 x 10^-8^ with SBP and DBP were extracted and clumped to a pairwise linkage disequilibrium (LD) threshold of r^2^ < 0.01 using PLINK v2.0 and phase 3 version 5 of 1000 Genomes Project European reference panel. Clumping is the process by which the variant with the strongest association per subset of variants in high LD with one another is selected. The number of instrumental variants, R^2^ values, F-statistics, and 1/F values for SBP and DBP are reported in Table S2. The R^2^ value quantifies the proportion of variance in the exposure explained by the genetic instrument. The F-statistic quantifies the strength of the relationship between the genetic instrument and the exposure. An F-statistic >10 indicates a low risk of weak instrument bias ^[36]^ and the expected relative magnitude of weak instrument bias can be approximated as 1/F^[36]^.

### Univariable Mendelian randomization

Mendelian randomization leverages genetic variants as instruments within an instrumental variable (IV) framework and rests on three core assumptions. First, the genetic instrument is robustly associated with the exposure. Second, the genetic instrument shares no common cause with the outcome. Third, the genetic instrument influences the outcome solely via the exposure. Violation of this third assumption is called ‘horizontal pleiotropy’. First, univariable MR was performed, wherein the ‘total’ effect of a single exposure on an outcome is estimated. Genetic associations were harmonised by aligning effect alleles in both exposure and outcome datasets, with no exclusions made for palindromic variants. MR estimates were generated by first calculating the Wald ratio for each variant, i.e., variant-outcome association divided by the variant-exposure association, before pooling these Wald estimates via the inverse-variance weighted (IVW) approach ^[37]^. MR estimates represent the change in IOP, mRNFL thickness, GCC thickness, VCDR, and odds ratio for POAG, per 10mmHg increase in SBP or DBP. MR analyses were performed using the *TwoSampleMR, MendelianRandomization, MR-PRESSO* and *MVMR* packages in R (version 4.1.2).

### Sensitivity analyses for pleiotropy, winner’s curse, and collider bias

The IVW approach assumes no horizontal pleiotropy and so a series of sensitivity analyses, including weighted median, contamination mixture, MR-Egger and MR-PRESSO methods were used to interrogate the robustness of results to horizontal pleiotropy. Further details of these methods can be found in the Supplementary Methods.

Evangelou *et al.’s* 2018 GWAS of blood pressure traits adjusted for BMI and Han *et al.’s* 2021 GWAS of VCDR adjusted for vertical disc diameter. Heritable covariable-adjusted GWAS data can introduce collider bias into subsequent MR analyses leveraging these genetic data ^[38]^. The resulting bias can be either toward or away from the null, with the direction of bias being unknown. We therefore performed a sensitivity analysis using Elsworth *et al.’s* UKBB GWAS of SBP and DBP, which did not adjust for BMI ^[39]^, and used Springelkamp *et al.’s* GWAS for VCDR, which did not adjust for disc diameter.

Winner’s curse is a phenomenon where genetic association estimates calculated using discovery GWAS datasets are exaggerated away from the null ^[40]^. Bias due to winner’s curse in gene-exposure estimates will lead to a deflation in MR effect estimates whereas bias due to winner’s curse in gene-outcome estimates will lead to an inflation in MR effect estimates. We performed a sensitivity analysis for any MR associations identified using instrumental variants at *P* < 10^-11^, which empirical studies have shown mitigate the impact of bias due to winner’s curse in MR estimates ^[40]^.

### Multivariable Mendelian randomization

Multivariable Mendelian randomization (MVMR) can be used to conduct formal mediation analyses within the MR framework by decomposing the ‘total’ effect of an exposure on an outcome - as estimated by univariable MR - into that which acts ‘directly’ on the outcome and that which act ‘indirectly’ on the outcome through proposed mediators ^[41]^. MVMR mediation analysis was therefore performed to investigate the effect of SBP acting ‘directly’ on mRNFL and GCC thickness (i.e., independent of IOP) vs that mediated ‘indirectly’ through changes in IOP. Specifically, in an MVMR model including both SBP and IOP as exposures, the MVMR estimate for SBP represents the effect of SBP whilst holding IOP constant, yielding the ‘direct’ effect of SBP on mRNFL and/or GCC thickness. Equivalence between the total and direct estimates of SBP on mRNFL and/or GCC thickness indicates a lack of mediation by IOP. Conversely, attenuation in the direct estimate for SBP as compared with the total estimate for SBP indicates mediation by IOP, with the degree of attenuation proportional to the total effect of SBP mediated through IOP. Variant-outcome associations (mRNFL and GCC thickness) were regressed on the variant-exposure (SBP) and variant-mediator (IOP) associations, with estimates weighted by the inverse of the standard error of variant-outcome associations and the intercept constrained at the origin ^[42]^. Q_het_ MVMR was conducted as a sensitivity analysis robust to conditionally weak instruments and beta coefficients were compared with those in the standard IVW model ^[43]^. Estimates for the indirect effect and proportion mediated through IOP were calculated using MVMR and network (or ‘two-step’) MR (see Supplemental Methods) ^[41]^.

## Results

### Intraocular Pressure

**Figure 2:**
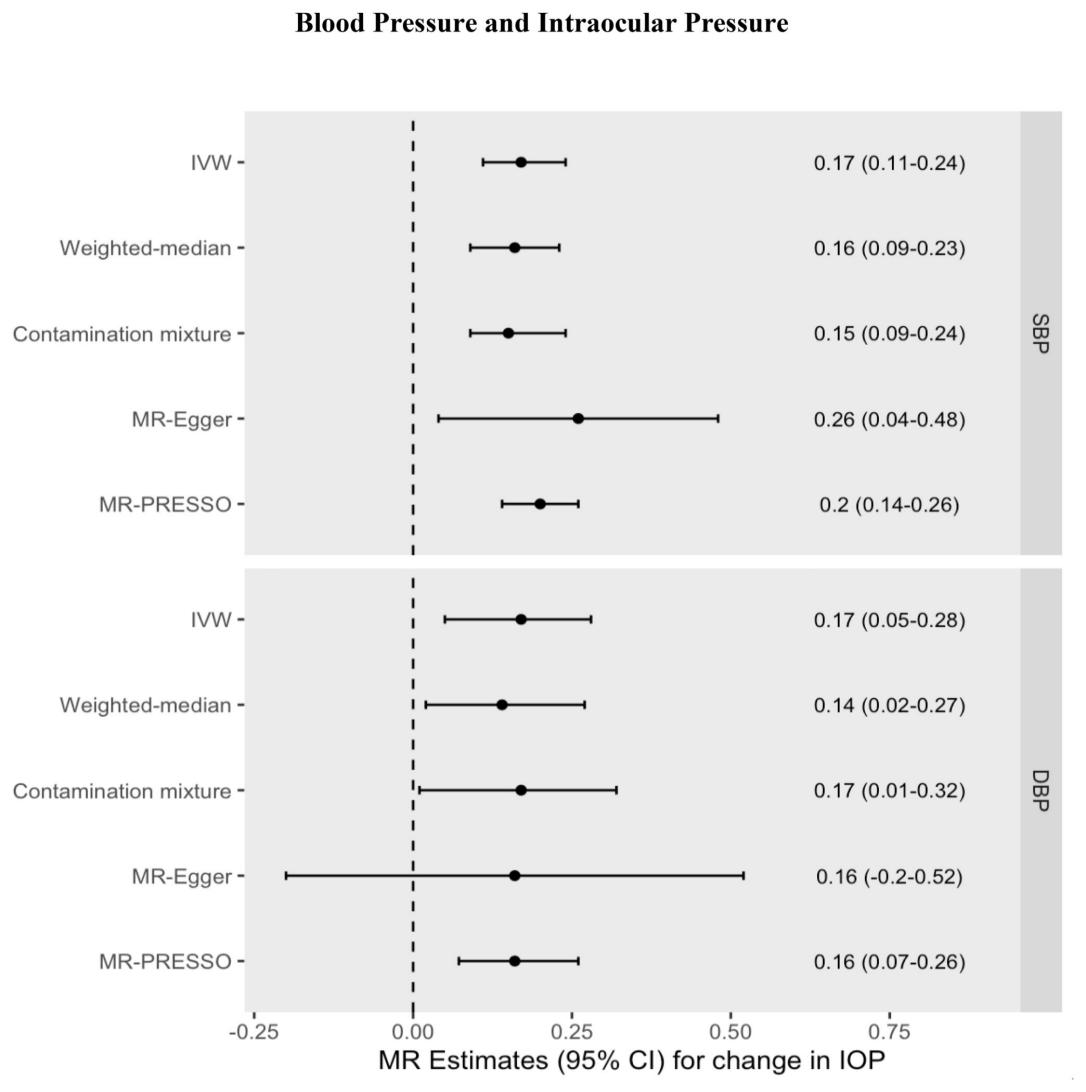
MR effect estimates (beta coefficients) for the change in IOP (mmHg) per 10mmHg increase in Systolic Blood Pressure (SBP) or Diastolic Blood Pressure (DBP). Primary estimate is the IVW = inverse variance-weighted. Pleiotropy-robust methods include MR-Egger, MR-PRESSO (Pleiotropy RESidual Sum and Outlier), Contamination mixture and Weighted-median methods.

Increased genetically predicted blood pressure was associated with a 0.17mmHg higher IOP per 10mmHg increase in SBP (95% CI=0.11 to 0.24mmHg increase in IOP, *P*=5.18 x 10^-7^) and a 0.17mmHg higher IOP per 10mmHg increase in DBP (95% CI=0.05 to 0.28mmHg increase in IOP, *P*=0.004). Results were consistent across pleiotropy-robust sensitivity analyses (see Figure 2, Tables S3-S4, Figures S1-S2). The MR-Egger intercept test did not identify any evidence of horizontal pleiotropy. The MR-PRESSO Global Heterogeneity test indicated the presence of horizontal pleiotropy, but after removal of potentially pleiotropic outliers, MR-PRESSO estimates remained consistent.

### Macular retinal nerve fiber layer thickness

**Figure 3:**
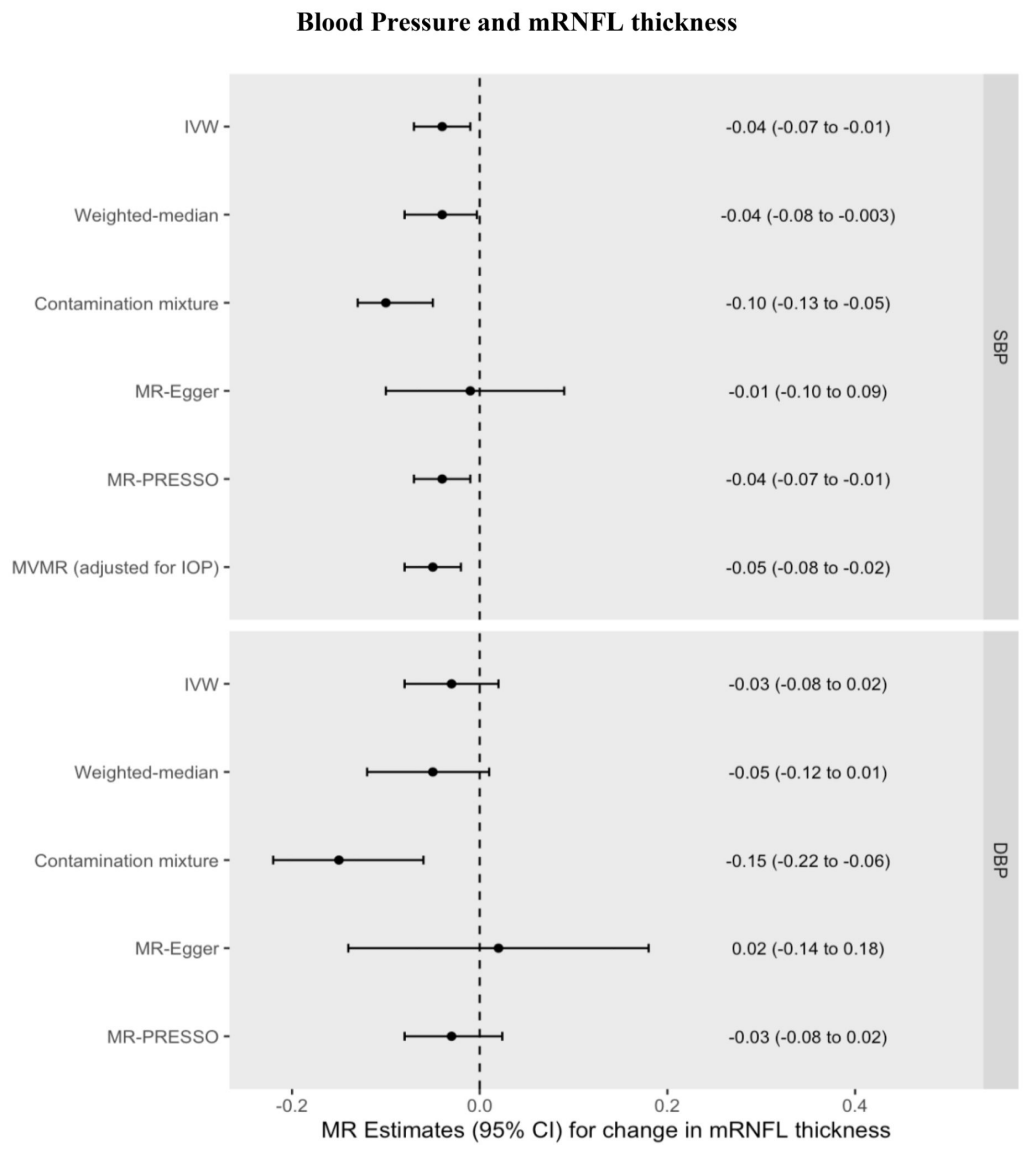
MR effect estimates (beta coefficients) for the change in mRNFL (µm) per 10mmHg increase in Systolic Blood Pressure (SBP) or Diastolic Blood Pressure (DBP). Primary estimate is the IVW = inverse variance-weighted. Pleiotropy-robust methods include MR-Egger, MR-PRESSO (Pleiotropy RESidual Sum and Outlier), Contamination mixture and Weighted-median methods. MVMR = multivariable MR.

Increased genetically predicted SBP was associated with a 0.04µm thinner mRNFL (95% CI=-0.07 to -0.01µm, *P*=0.004) per 10mmHg increase. Increased genetically predicted DBP was associated with a 0.03µm thinner mRNFL (95% CI=-0.08 to 0.02µm per 10mmHg increase, *P*=0.257). These univariable MR results were consistent across pleiotropy-robust sensitivity analyses (see Table S5, Figure 3, Figures S3-S4). Multivariable MR found that the direct effect of SBP on mRNFL thickness after adjusting for IOP (0.05µm decrease, 95% CI=-0.08 to -0.02µm, *P*=0.002) was virtually unchanged as compared with the univariable MR estimate of the total effect, indicating a lack of mediation by IOP. The Q_het_ MVMR model adjusting for conditionally weak instruments (F-statistic for IOP=2.3) yielded a similar beta coefficient = -0.04µm. Using MVMR, the indirect effect of SBP on mRNFL thickness mediated through IOP was 0.002µm (95% CI=-0.003µm to 0.005µm), corresponding to a proportion mediated of -3% (95% CI=-14% to 8%). Using network MR, the estimates were similar with the indirect effect calculated as 0.001µm (95% CI=-0.004µm to 0.007µm) and the proportion mediated through IOP = -3% (95% CI=-19% to 9%).

### Ganglion cell complex thickness

**Figure 4:**
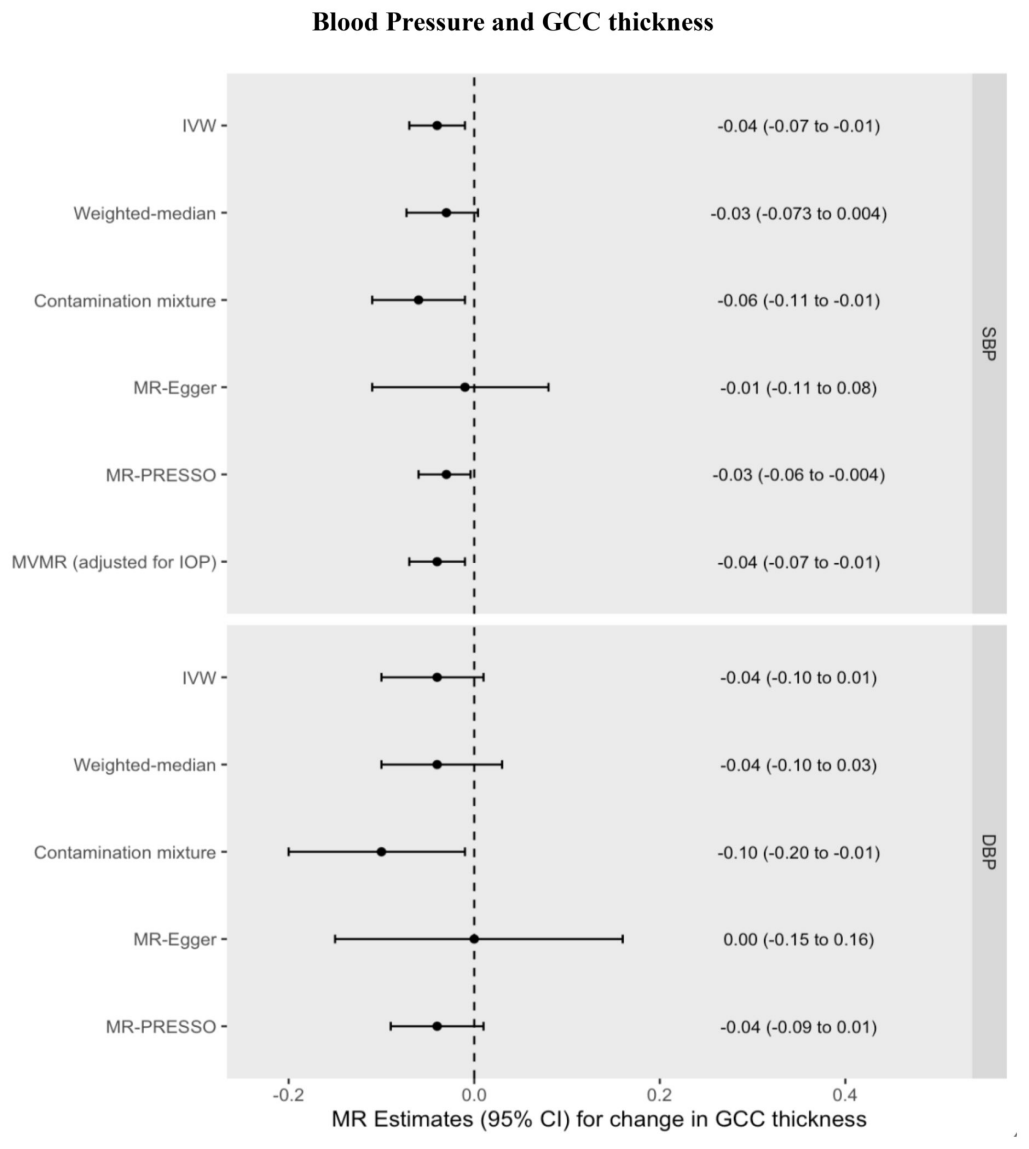
MR effect estimates (beta coefficients) for the change in GCC (µm) per 10mmHg increase in Systolic Blood Pressure (SBP) or Diastolic Blood Pressure (DBP). Primary estimate is the IVW = inverse variance-weighted. Pleiotropy-robust methods include MR-Egger, MR-PRESSO (Pleiotropy RESidual Sum and Outlier), Contamination mixture and Weighted-median methods. MVMR = multivariable MR.

Increased genetically predicted SBP was also associated with a 0.04µm thinner GCC (95% CI=-0.07 to -0.01µm, *P*=0.018) per 10mmHg increase in SBP. Increased genetically predicted DBP was associated with a 0.04µm thinner GCC (95% CI=-0.10µm to 0.01µm per 10mmHg increase, *P*=0.121). These univariable MR results were consistent across pleiotropy-robust sensitivity analyses (see Table S6, Figure 4, Figures S5-S6). The MVMR estimate for the direct effect of SBP on GCC thickness (0.04µm decrease, 95% CI=-0.07 to -0.01µm, *P*=0.01) was unchanged with respect to the univariable MR estimate of the total effect, again indicating a lack of mediation by IOP. This result was robust to the conditionally weak instrument for IOP (Q_het_ MVMR model beta coefficient = -0.04µm). Using MVMR, the indirect effect of SBP on GCC thickness mediated through IOP was 0.0005µm (95% CI=-0.004µm to 0.004µm), corresponding to a proportion mediated of -1% (95% CI=-15% to 13%). Using network MR, the estimates remained virtually unchanged with an indirect effect of 0.0005µm (95% CI=-0.005µm to 0.006µm) and the proportion mediated through IOP = -1% (95% CI=-6% to 6%).

### Primary open-angle glaucoma

**Figure 5:**
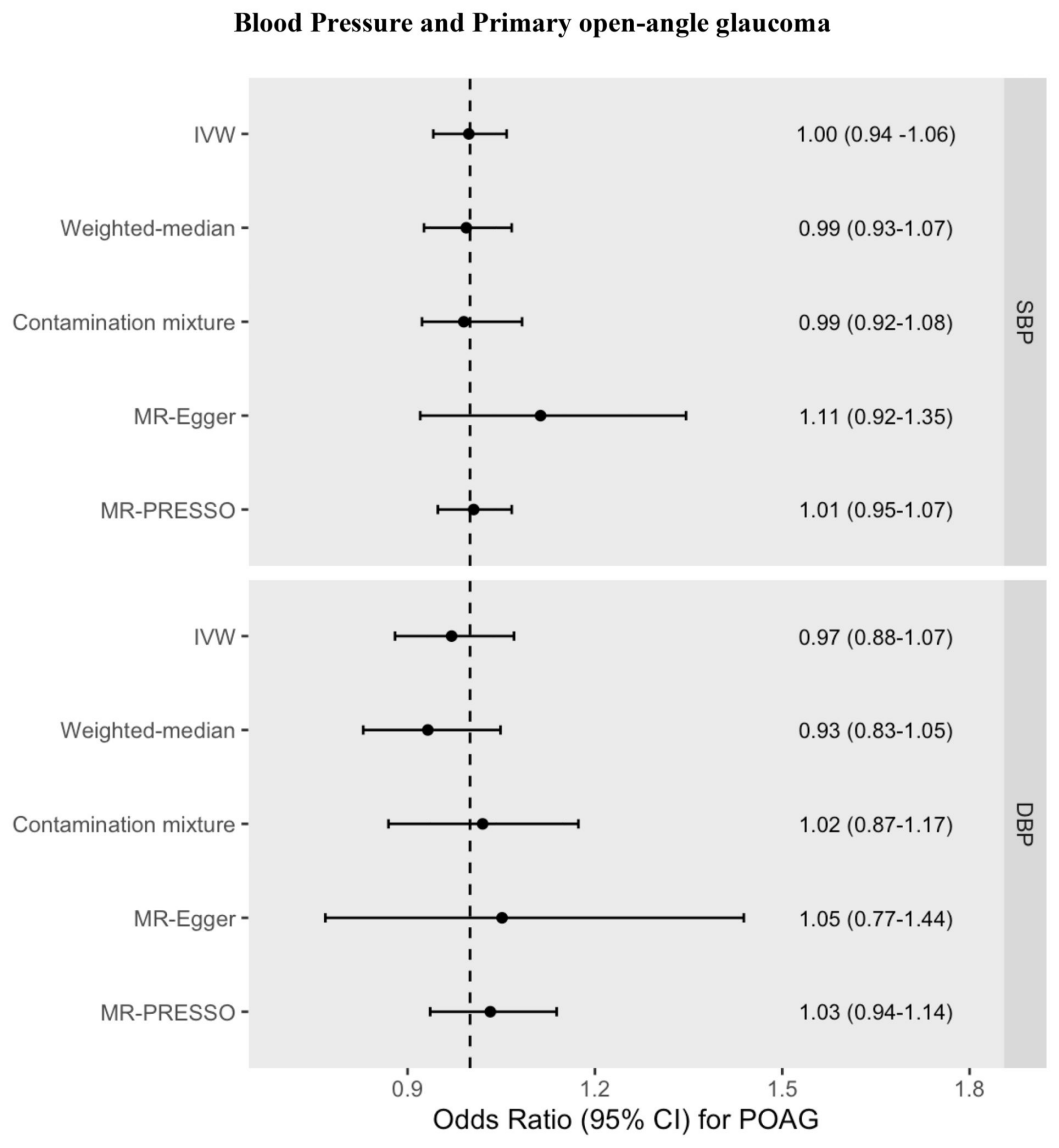
MR effect estimates (Odds ratios (ORs) for POAG liability per 10mmHg increase in Systolic Blood Pressure (SBP) or Diastolic Blood Pressure (DBP). OR was calculated by taking the exponential of the IVW beta coefficient in the logistic regression equation. Pleiotropy-robust methods include MR-Egger, MR-PRESSO (Pleiotropy RESidual Sum and Outlier), Contamination mixture and Weighted-median methods.

Neither genetically predicted SBP nor DBP associated with liability to POAG (OR for SBP = 1.00 95% CI=0.94 to 1.06, *P*=0.951, and OR for DBP = 0.97, 95% CI=0.88 to 1.07, *P*=0.591) (see Tables S3-S4, Figure 5, Figures S7-S8).

### Vertical cup-to-disc ratio

Neither genetically predicted SBP nor DBP associated with VCDR (0.0004, 95% CI=-0.002 to 0.003, *P*=0.760 for SBP, and -0.001, 95% CI=-0.004 to 0.002, *P*=0.544, for DBP) (see Tables S11-S12, Figures S9-S11). This null result was replicated in a smaller GWAS for VCDR where no adjustment for disc diameter was made (Tables S13-S14).

### Additional Sensitivity Analyses

Sensitivity analyses indicate that MR associations for IOP, mRNFL thickness, and GCC thickness were robust to winner’s curse and collider bias owing to the adjustment for BMI in SBP and DBP GWASs (see Tables S3-S10).

## Discussion

### Principal findings

We find genetic evidence in support of a causal effect of lifelong elevated systolic and diastolic blood pressure on increased intraocular pressure, and of elevated systolic blood pressure on both mRNFL and GCC thinning. Interestingly, MR mediation analysis supports an effect of elevated systolic blood pressure on retinal ganglion cell degeneration arising independently of intraocular pressure. In turn, these findings support the importance of blood pressure as a modifiable risk factor in the development of increased intraocular pressure and, independently, in retinal ganglion cell degeneration.

### Blood pressure and intraocular pressure

A range of mechanisms have been hypothesized to explain the link between blood pressure and IOP. Elevated blood pressure may increase ciliary perfusion pressures and lead to greater ultrafiltration of aqueous fluid in the ciliary body ^[44]^. Increased blood pressure may also increase episcleral venous pressures and reduce aqueous humour outflow ^[44]^. Numerous previous observational studies identify a positive association between both SBP and DBP and IOP ^[13] [14] [15]^. We leveraged the largest published GWASs of SBP, DBP, and IOP to maximise statistical power, which likely explains why previous MR studies did not detect an effect of either SBP or DBP on IOP ^[45]^. The F-statistics and 1/F values reported in Table S2 show a low risk of weak instrument bias and so the presence of sample overlap between the exposures and outcomes is highly unlikely to materially impact our results ^[36]^. Clinically, increases in IOP do not necessarily lead to ocular hypertension and not every individual with ocular hypertension necessarily develops POAG ^[9]^. However, given prior evidence demonstrating that lowering IOP from any baseline level slows disease progression in patients with POAG ^[9]^, the effect of blood pressure on IOP may be of particular clinical relevance in those individuals with, or at high risk of developing, POAG.

### Blood pressure and retinal ganglion cell degeneration

We found concordant genetic associations between SBP and both mRNFL and GCC thickness, two biomarkers widely used to detect and monitor glaucomatous changes in clinical practice. In the largest traditional observational analysis conducted to date, Huang *et al*. found that elevated SBP and DBP associated with a thinner macular ganglion cell-inner plexiform layer (mGCIPL) thickness and thinner mRNFL in approximately 23,000 individuals from the UKBB and Chinese Ocular Imaging project (COIP) ^[16]^. In a longitudinal analysis involving over 2000 individuals in COIP, they found that elevated SBP and DBP associated with a faster rate of mGCIPL thinning and elevated SBP associated with a faster rate of circumpapillary RNFL (cRNFL) thinning. Of note, they found that the effect of blood pressure on mRNFL thinning and cRNFL thinning was concordant. These findings are consistent with numerous other studies ^[46] [17] [18]^. For instance, Marshall *et al*. found that systemic hypertension was associated with longitudinal mGCIPL and cRNFL thinning and visual decline in a prospective cohort of 1,314 stable glaucoma patients ^[46]^.

However, other studies have produced conflicting results ^[19] [47]^. One recent longitudinal study did not detect any association between long-term elevated blood pressure and either mRNFL or GCC thickness ^[19]^. In this study, elevated blood pressure later in life (mean age of 74.2 to 79.1 years) was defined as having either elevated systolic or diastolic blood pressure or antihypertensive use in the previous 2 weeks. Consequently, a proportion of patients defined as hypertensive but who were on antihypertensive medication may actually have had blood pressures within the normal range, and the potentially weaker or absent effect of DBP may have nullified any effect of SBP to produce an overall null result. Given the sample size of approximately 900 individuals in the primary analysis, the study is also limited in power. In our study, the similar point estimates yet wider confidence intervals for DBP as those seen for SBP for both mRNFL and GCC thickness (Figures 3 and 4) raise the possibility that the lack of an observed effect for DBP reflects insufficient statistical power. However, an association between low DBP and increased glaucoma risk is well-established in the epidemiological literature ^[48] [49] [50]^ and a recent longitudinal study containing 105 POAG patients found that a combination of higher IOP and lower baseline diastolic blood pressure associated with a faster rate of GCC thinning ^[47]^. Longitudinal GWAS data leveraged in the MR framework could help investigate whether previously reported associations between DBP and rate of GCC thinning over time are indeed causal.

Given the conventional observational designs of all previous studies on this question, such data are inherently vulnerable to unmeasured confounding. We leveraged MR to strengthen robustness to unmeasured confounding and investigate the causal influence of blood pressure on OCT-derived biomarkers of retinal ganglion cell loss. Sensitivity analyses suggest that MR associations for IOP, RNFL thickness, and GCC thickness, were robust to the potential influence of horizontal pleiotropy, winner’s curse, and collider bias, thus strengthening causal inference. Moreover, upon adjustment for genetically predicted IOP in our MVMR model, there was no attenuation of the genetic association between SBP on either mRNFL or GCC thickness with respect to the univariable MR estimate. Furthermore, two different MR mediation analyses estimated that the proportion of the effect of SBP on mRNFL thickness and GCC thickness mediated through IOP was -3% and -1%, respectively. Although the 95% confidence intervals remain consistent with a small degree of mediation, and the absence of any mediation whatsoever cannot definitively be proven, these results suggest a lack of mediation by IOP. Consistent with our findings, previous studies have shown that the association between SBP and mGCIPL progression is independent of IOP ^[46]^. This implies that the effect of blood pressure on retinal ganglion cell degeneration is mediated by biological pathways distinct from elevated IOP. POAG is known to arise across a spectrum of IOP, including within the normal range, and vascular dysfunction, e.g., endothelial dysfunction and impaired autoregulatory reserve, is one proposed category of IOP-independent mechanisms in the pathogenesis of POAG ^[51]^. Mechanistic studies will be important in exploring whether such vascular dysfunction mediates the observed effect of SBP on retinal ganglion cell degeneration or whether distinct biological mechanisms are involved.

### Blood pressure, VCDR and primary open-angle glaucoma

In this study, we did not find a genetic association between blood pressure and either VCDR or liability to POAG ^[45]^. This is in line with a previous MR analysis of the effect of blood pressure on POAG liability and indeed, the association between blood pressure on POAG risk is inconsistent in the epidemiological literature ^[52] [53]^. The null result for POAG may in part reflect reduced statistical power due to both the binary nature of the outcome data and the presence of clinical heterogeneity amongst cases in the original GWAS. The inclusion of a small number of patients with normal-tension glaucoma in the POAG GWAS where low rather than high blood pressure may be more important in disease pathogenesis ^[54]^ could partly nullify the genetic association. Indeed, an advantage of leveraging continuous glaucoma-related outcome traits such as RNFL thickness, GCC thickness, and VCDR, is greater statistical power for identifying smaller associations and an increased robustness to misclassification bias. VCDR may also be a more specific marker of glaucoma than mRNFL thickness and GCC thickness ^[55] [56]^. Thus, the null result for VCDR supports the null result for POAG liability, and taken together with the associations of systolic blood pressure with IOP, mRNFL thickness, and GCC thickness, these findings point to a complex and at times conflicting effect of blood pressure in the setting of glaucoma.

Differences in the overall effect of blood pressure on glaucoma endophenotypes vs liability to POAG itself is well described in the literature. A large-scale meta-analysis showed that whilst virtually all studies found a positive association between SBP, DBP and IOP, there was significant heterogeneity in the relationship between blood pressure and POAG, with 18 studies reporting a positive association whilst 9 reported an inverse or null association ^[52]^. Therefore, another possible explanation for our null results is a U-shaped relationship between blood pressure on POAG liability, where risk is increased for those with very low or very high blood pressure. U-shaped associations with glaucoma have been reported for both SBP ^[57]^ and DBP ^[47]^, even in those not on antihypertensive medications ^[58]^. Similarly, both low ^[20] [59]^ and high blood pressure ^[60] [61] [21]^ have been reported to associate with increased VCDR, though to our knowledge no studies have directly investigated the association of blood pressure with VCDR using non-linear models. Whilst methods for non-linear MR have been developed ^[62]^, large-scale individual participant data in a one-sample setting is required and such data are not currently available for POAG or VCDR.

### Limitations

Our study has limitations. Firstly, given the absence of genetic association data available for circumpapillary RNFL (cRNFL) thickness, the present study investigated mRNFL thickness. Prior studies suggest that cRNFL thickness may be a more accurate biomarker for early glaucomatous damage than mRNFL thickness ^[63]^, however, the two are strongly correlated and mRNFL thickness has itself been shown to identify early glaucomatous visual field defects ^[7] [8]^. Similarly, the GCIPL was not separately segmented in Zekavat *et al*.’s GWAS but further studies could examine the extent to which the result for GCC thickness was driven by the result for mRNFL thickness or due to independent thinning of the GCIPL. Secondly, although widely considered a sensitive and clinically relevant biomarker early glaucoma, mRNFL thinning is not specific to glaucoma and rather, it may serve as marker of retinal neurodegenerative processes that are common to various diseases ^[64]^. GCC thinning has also recently been shown to associate with multiple sclerosis, alcohol use disorder, heart failure, and aortic aneurysms development ^[30]^. The influence of blood pressure on these retinal parameters may therefore not be specific to POAG and may have biological or clinical implications beyond the scope of POAG and indeed, ocular disease in general. Thirdly, MR point estimates reflect the effect of small lifelong differences in genetic liability to increased blood pressure and so they are not readily interpretable on the same scale as an equivalent clinical intervention, i.e., estimates from a randomized controlled trial investigating the IOP-lowering effect of antihypertensive medications. Finally, given that the frequency and distribution of genetic variants differ across ancestries, we restricted our analysis to European ancestry individuals to avoid confounding by ancestry. Consequently, these findings may not be generalisable to other ancestries.

## Conclusions

Mendelian randomization analysis supports a causal effect of lifelong elevated blood pressure on increased IOP and retinal ganglion cell degeneration, though the effect on liability to POAG remains uncertain. Interestingly, MR mediation analysis supports an effect of elevated systolic blood pressure on retinal ganglion cell degeneration that arises independently of IOP. This implies that targeted blood pressure control, for instance through lifestyle modification and antihypertensive medication, could help preserve vision by lowering IOP and by preventing retinal ganglion cell degeneration, including in individuals with a normal eye pressure.

## Supplementary Material

Supplementary material

Supplementary methods
