# Supplementary material for: Independent Effects of Blood Pressure on Intraocular Pressure and Retinal Ganglion Cell Degeneration: A Mendelian randomization study
Source: Invest Ophthalmol Vis Sci. Author manuscript; Available in PMC 2024 Jul 22. (PMC11262474; doi:10.1167/iovs.65.8.35)
Supplement: Supplementary methods [file EMS197625-supplement-Supplementary_methods.docx]

**Supplementary Methods**

**Pleiotropy-robust Sensitivity Analyses**

The presence of horizontal pleiotropy, whereby the genetic instrument influences the outcome not solely via the exposure, i.e., systemic blood pressure, constitutes a violation of the third MR assumption. The IVW approach provides a valid causal estimate under the assumption that all variants are valid instrumental variables ^[1, 2]^. This assumes that there is no horizontal pleiotropy, or that pleiotropy is balanced (pleiotropy may be present in opposing directions but its averages out at zero). Even though the absence of horizontal pleiotropy cannot definitively be proven in any given MR analysis, sensitivity analyses can be used to interrogate the robustness of results to such pleiotropy.

In median-based methods, a median rather than mean of the Wald estimates is calculated. Provided that over 50% of the weights are come from valid instruments, median-based estimates will produce valid causal estimates. In the simple-median approach ^[3]^, all variant-specific estimates are weighted equally, whereas in the weighted-median approach ^[3]^, variants are weighted inversely to their standard errors.

The contamination mixture method provides valid causal estimates under the plurality valid assumption, i.e., that a plurality of the genetic variants are valid instruments ^[4]^. A likelihood function is constructed from the variant-specific ratio estimates. If a variant is a valid instrument, then its ratio estimate is assumed to be normally distributed about the true causal effect. If a variant is an invalid instrument, then its ratio estimate is assumed to be distributed about 0 with a large standard deviation. The likelihood is then maximised over different values of the causal effect and different configurations of valid and invalid instruments. The confidence intervals are usually not symmetric. Default parameters were used in the analysis.

Rather than fixing the intercept at the origin, as done in the IVW approach, MR-Egger ^[5]^ allows the intercept to float and provides a valid causal estimate in the presence of directional pleiotropy. MR-Egger requires the instrument strength independent of the direct effects (INSIDE) assumption, i.e., it requires that the pleiotropic effects of instrumental variants are independent of the association between the variants and the exposure. The precision of MR-Egger depends on the variance between genetic associations with the exposure and so if the different instrumental variants are similarly associated with the exposure, MR-Egger will be imprecise and have wide confidence intervals. Indeed, of the different sensitivity analyses used, MR-Egger can be expected to have the widest confidence intervals. The deviation of the intercept term from the origin can be tested to indicate whether a significant degree of directional pleiotropy is present.

The MR-Pleiotropy Residual Sum and Outlier (MR-PRESSO) method ^[6]^ takes the residual sum of squares from the IVW linear regression equation as a measure of heterogeneity. The IVW method is performed iteratively, removing each instrumental variant in turn, and the RSS calculated for each omission. Significantly heterogenous variants are removed and the IVW method used to calculate the MR estimate using the remaining variants.

**MR Mediation Analyses**

Two MR approaches, multivariable MR (MVMR) and network (or ‘two-step’), can be used to calculate the indirect effect of an exposure on an outcome and the proportion of this effect mediated through a proposed mediator ^[7]^. Using MVMR, the indirect effect equates to the difference between the total and direct effects of the exposure on the outcome as derived from univariable MR and MVMR, respectively, i.e., the indirect effect = total effect – direct effect. The proportion mediated is then calculated as the indirect effect expressed as a proportion of the total effect, i.e. (total effect – direct effect)/ total effect. The MVMR approach is equivalent to the ‘difference method’ in traditional mediation analysis. In network (or ‘two-step’), the mediated effect is calculated by multiplying the effect of the exposure on the mediator by the effect of the mediator on the outcome, and the proportion mediated is then calculated as mediated effect/ total effect. The network MR approach is equivalent to the ‘product of coefficients’ method in traditional mediation analysis.

# References

| [1] | Burgess S, Small DS, Thompson SG. A review of instrumental variable estimators for Mendelian randomization. Stat Methods Med Res. 2017;26(5):2333-2355. |
| --- | --- |
| [2] | Burgess S, Butterworth A, Thompson SG. Mendelian randomization analysis with multiple genetic variants using summarized data. Genet Epidemiol. 2013;37(7):658-665. |
| [3] | Bowden J, Davey Smith G, Haycock PC, Burgess S. Consistent Estimation in Mendelian Randomization with Some Invalid Instruments Using a Weighted Median Estimator. Genet Epidemiol. 2016;40(4):304-314. |
| [4] | Burgess S, Foley CN, Allara E, Staley JR, Howson JMM. A robust and efficient method for Mendelian randomization with hundreds of genetic variants. Nat Commun. 2020;11(1):376. |
| [5] | Bowden J, Davey Smith G, Burgess S. Mendelian randomization with invalid instruments: effect estimation and bias detection through Egger regression. Int J Epidemiol. 2015;44(2):512-525. |
| [6] | Verbanck M, Chen CY, Neale B, Do R. Detection of widespread horizontal pleiotropy in causal relationships inferred from Mendelian randomization between complex traits and diseases. Nat Genet. 2018;50(5):693-698. |
| [7] | Sanderson E. Multivariable Mendelian Randomization and Mediation. Cold Spring Harb Perspect Med. 2021;11(2):a038984. |
