# Supplementary material for: Independent Effects of Blood Pressure on Intraocular Pressure and Retinal Ganglion Cell Degeneration: A Mendelian Randomization Study
Source: Invest Ophthalmol Vis Sci. 2024 Jul 19;65(8):35. doi: 10.1167/iovs.65.8.35 (PMC11262474; doi:10.1167/iovs.65.8.35)
Supplement: Supplementary file 1 [file iovs-65-8-35_s001.pdf]

Supplementary Materials

Table S1 - Summary of data sources

Table S2 - The number of instrumental variants, R^2 values & F-statistics for SBP & DBP

Table S3 - Mendelian randomization estimates for the effect of genetically predicted Systolic Blood Pressure (SBP) (Evangelou *et al.'s* combined ICBP & UKBB GWAS) on intraocular pressure (IOP) and primary open-angle glaucoma (POAG)

Table S4 - Mendelian randomization estimates for the effect of genetically predicted Diastolic Blood Pressure (DBP) (Evangelou *et al.*'s combined ICBP & UKBB GWAS) on intraocular pressure (IOP) and primary open-angle glaucoma (POAG)

Table S5 - Mendelian randomization estimates for the effect of genetically predicted SBP and DBP (Evangelou *et al.'s* combined ICBP & UKBB GWAS) on macular retinal nerve fiber layer (mRNFL) thickness

Table S6 - Mendelian randomization estimates for the effect of genetically predicted SBP and DBP (Evangelou *et al.'s* combined ICBP & UKBB GWAS) on ganglion cell complex (GCC) thickness

Table S7 - Mendelian randomization estimates for the effect of genetically predicted Systolic Blood Pressure (SBP) (Elsworth *et al.'s* UKBB 2018 GWAS) on intraocular pressure (IOP) and primary open-angle glaucoma (POAG)

Table S8 - Mendelian randomization estimates for the effect of genetically predicted Systolic Blood Pressure (SBP) and Diastolic Blood Pressure (DBP) (Elsworth *et al.'s* UKBB 2018 GWAS) on macular retinal nerve fiber layer thickness (mRNFL)

Table S9 - Mendelian randomization estimates for the effect of genetically predicted Systolic Blood Pressure (SBP) and Diastolic Blood Pressure (DBP) (Elsworth *et al.'s* UKBB 2018 GWAS) on ganglion cell complex (GCC) thickness

Table S10 - Mendelian randomization estimates for the effect of genetically predicted Diastolic Blood Pressure (DBP) (Elsworth *et al.*'s UKBB 2018 GWAS) on intraocular pressure (IOP) and primary open-angle glaucoma (POAG)

Table S11 - Mendelian randomization estimates for the effect of genetically predicted SBP and DBP (Evangelou *et al.'s* combined ICBP & UKBB GWAS) on vertical-cup disc ratio (VCDR) (Han *et al.* 2021)

Table S12 - Mendelian randomization estimates for the effect of genetically predicted SBP and DBP (Elsworth *et al.'s* UKBB 2018 GWAS) on vertical-cup disc ratio (VCDR) (Han *et al.* 2021)

Table S13 - Mendelian randomization estimates for the effect of genetically predicted SBP and DBP (Evangelou *et al.'s* combined ICBP & UKBB GWAS) on vertical-cup disc ratio (VCDR) (Springelkamp *et al.* 2017)

Table S14 - Mendelian randomization estimates for the effect of genetically predicted SBP and DBP (Elsworth *et al.'s* UKBB 2018 GWAS) on vertical-cup disc ratio (VCDR) (Springelkamp *et al.* 2017)

Figure S1 – Scatter plot of the genetic associations of instrumental variants with systolic blood pressure and intraocular pressure.

Figure S2 – Scatter plot of the genetic associations of instrumental variants with diastolic blood pressure and intraocular pressure.

Figure S3 – Scatter plot of the genetic associations of instrumental variants with systolic blood pressure and mRNFL thickness.

Figure S4 – Scatter plot of the genetic associations of instrumental variants with diastolic blood pressure and RNFL thickness.
Figure S5 – Scatter plot of the genetic associations of instrumental variants with systolic blood pressure and GCC thickness.
Figure S6 – Scatter plot of the genetic associations of instrumental variants with diastolic blood pressure and GCC thickness.
Figure S7 – Scatter plot of the genetic associations of instrumental variants with systolic blood pressure and primary open-angle glaucoma
Figure S8 – Scatter plot of the genetic associations of instrumental variants with diastolic blood pressure and primary open-angle glaucoma
Figure S9 – Forest plot of MR estimates for the effect of genetically predicted SBP and DBP on VCDR
Figure S10 – Scatter plot of the genetic associations of instrumental variants with systolic blood pressure and VCDR
Figure S11– Scatter plot of the genetic associations of instrumental variants with diastolic blood pressure and VCDR
Table S15 – STROBE-MR Checklist

Table S1 - Summary of data sources

Phenotype	Study	Ancestry	N cases (/ controls)	Description (including case definition & ascertainment)
Systolic Blood Pressure	Evangelou *et al.* 2018	EUR	757,601	Meta-analysis of 77 participating cohorts in the ICBP and the UKBB, making it the largest GWAS of SBP performed to date. SBP was measured using either manual or automated readings (mmHg) and an average of two or more readings were taken for the vast majority of participants. All cohorts adjusted for age, age^2, sex, and BMI.
Systolic Blood Pressure	Elsworth *et al.* 2018	EUR	436,419	Automated SBP readings were taken in UKBB participants
Diastolic Blood Pressure	Evangelou *et al.* 2018	EUR	757,601	Meta-analysis of 77 participating cohorts in the ICBP and the UKBB, making it the largest GWAS of DBP performed to date. DBP was measured using either manual or automated readings (mmHg) and an average of two or more readings were taken for the vast majority of participants. All cohorts adjusted for age, age^2, sex, and BMI.
Diastolic Blood Pressure	Elsworth *et al.* 2018	EUR	436,424	Automated DBP readings were taken in UKBB participants
Intraocular pressure	Khawaja *et al.* 2018	EUR	139,555	UK Biobank: IOP was measured once in each eye using the Ocular Response Analyzer (ORA, Reichert Inc.), a non-contact tonometer that measures the pressure on corneal flattening on both inward and outward motions, in response to a jet of air. These pressures were used to derive a Goldmann-correlated IOP (IOPg) and then corneal-compensated IOP (IOPcc), a measure of IOP least affected by corneal biomechanical properties.<br><br>EPIC Norfolk Eye Study: IOPcc was calculated as above for >94% participants.<br><br>IGGC: IOP was not corneal-compensated in all participating studies. Goldmann applanation tonometry was the most common method of IOP measurement but approaches varied across the different individual studies and details can be found in the Supplementary Methods in Springelkamp *et al.* 2017<br><br>Adjustments were made for age, sex, and the first five principal components. This is the largest GWAS of IOP performed to date.
Primary open-angle glaucoma	Gharahkhani *et al.* 2021	EUR	16,677/ 199,580	For most studies, POAG was defined according to International Classification of diseases (ICD)-9 and ICD-10 criteria. In conducting the GWAS, adjustments were made for age, sex, and study-specific principal components as covariates. Full details of case definitions can be found in the Supplementary Information of Gharahkhani *et al.* 2021. This is the largest publicly available GWAS of POAG performed to date.

Macular Retinal Nerve Fiber Layer (mRNFL) thickness<br><br>Ganglion Cell Complex (GCC) thickness	Zekavat *et al.* 2024	EUR	33,129	Nonmydriatic spectral domain OCT scans of the macula were obtained using Spectral Domain Topcon 3D OCT 1000 Mark II (Topcon GB, Newberry, Berkshire, UK). Three-dimensional 6x6 mm^2 macular volume scans were obtained (512 horizontal A-scans per B-scan: 128 B-scans in a 6x6 mm raster pattern). The right eye of each participant was imaged first, followed by the left eye. All OCT images were stored as .fds image files without prior analysis of macular thickness.<br><br>The Topcon Advanced Boundary Segmentation (TABS) algorithm was used to automatically segment all scans. This uses dual-scale gradient information to allow for automated segmentation of the inner and outer retinal boundaries and retinal sublayers.<br><br>All images with image quality scores less than 40 and images representing the poorest 10% as designated by the ILM indicator were excluded. Any image with a layer thickness greater than 2.5 standard deviations away from the mean was also excluded.<br><br>In conducting the GWAS, adjustments were made for age, age^2, sex, smoking, spherical equivalent, the first ten principal components of genetic ancestry, and genotyping array. This is the largest GWAS of mRNFL and GCC performed to date.
VCDR (adjusted for optic disc diameter)	Han *et al.* 2021	EUR	111,724	Convolutional neural networks (CNN) with a ResNet-34 architecture were trained for image gradeability, VCDR, and vertical disc diameter (VDD) based on ~70,000 fundus images in the UKBB. These models were then applied to the all UKBB fundus images (175,770 images) and another independent cohort, the Canadian Longitudinal Study on Aging (CLSA) (106,330 images). CNN models were used to grade left and right images from two visits for all participants.<br><br>In the UKBB, fundus images were obtained with the Topcon 3D OCT 1000 Mk2 instrument. In the CLSA, fundus images were obtained with the Topcon (TRC-NW8) non mydriatic retinal camera. In the IGGC, fundus images were obtained with different instruments across the 18 constituent cohorts, details of which can be found in Springelkamp *et al.*'s Supplement.<br><br>AI-based GWASs were performed for VCDR adjusted for VDD and genetic discoveries were replicated in clinician-graded fundus images from the International Glaucoma Genetics Consortium (IGGC). In conducting the GWAS, adjustments were made for sex, age and the first ten principal components. This is the largest GWAS of VCDR performed to date.
VCDR	Springelkamp *et al.* 2017	EUR	23,899	GWAS meta-analysis of 19 participating cohorts in the IGCC, of which 18 contained data on VCDR. Genotyping and phenotyping methods for each of these studies can be found in the Supplement of Springelkamp *et al.* 2017. Analyses were adjusted for age, sex, and the first 5 principal components of genetic ancestry (for population-based studied) or family structure (for family-based studies).

Table S2 - The number of instrumental variants, R^2 values & F-statistics for SBP & DBP

Exposure GWAS	P-value and LD clumping threshold	Exposure	Outcome	No. of instrumental SNPs	R^2	F-statistic	1/F
Combined ICBP and UKBB GWAS	$P < 5e-8$ & $r^2 < 0.01$	SBP	IOP	918	4.7%	40.7	2.5%
Combined ICBP and UKBB GWAS	$P < 1e-11$ & $r^2 < 0.01$	SBP	IOP	419	3.2%	60.1	1.7%
UKBB GWAS	$P < 5e-8$ & $r^2 < 0.01$	SBP	IOP	316	4.1%	58.3	1.7%
Combined ICBP and UKBB GWAS	$P < 5e-8$ & $r^2 < 0.01$	SBP	POAG	926	4.7%	40.7	2.5%
UKBB GWAS	$P < 5e-8$ & $r^2 < 0.01$	SBP	POAG	318	4.1%	58.4	1.7%
Combined ICBP and UKBB GWAS	$P < 5e-8$ & $r^2 < 0.01$	SBP	VCDR	884	4.4%	39.1	2.6%
UKBB GWAS	$P < 5e-8$ & $r^2 < 0.01$	SBP	VCDR	297	3.5%	53.1	1.9%
Combined ICBP and UKBB GWAS	$P < 5e-8$ & $r^2 < 0.01$	SBP	RNFL	926	4.7%	40.7	2.5%
Combined ICBP and UKBB GWAS	$P < 1e-11$ & $r^2 < 0.01$	SBP	RNFL	424	3.3%	60.0	1.7%
Combined ICBP and UKBB GWAS	$P < 5e-8$ & $r^2 < 0.01$	SBP	GCC	926	4.7%	40.7	2.5%
Combined ICBP and UKBB GWAS	$P < 1e-11$ & $r^2 < 0.01$	SBP	GCC	424	3.3%	60.0	1.7%
Combined ICBP and UKBB GWAS	$P < 5e-8$ & $r^2 < 0.01$	DBP	IOP	971	5.0%	40.8	2.5%
Combined ICBP and UKBB GWAS	$P < 1e-11$ & $r^2 < 0.01$	DBP	IOP	433	3.4%	61.5	1.6%
UKBB GWAS	$P < 5e-8$ & $r^2 < 0.01$	DBP	IOP	307	4.4%	65.3	1.5%
Combined ICBP and UKBB GWAS	$P < 5e-8$ & $r^2 < 0.01$	DBP	POAG	984	5.1%	40.9	2.4%
UKBB GWAS	$P < 5e-8$ & $r^2 < 0.01$	DBP	POAG	310	4.4%	65.4	1.5%
Combined ICBP and UKBB GWAS	$P < 5e-8$ & $r^2 < 0.01$	DBP	VCDR	920	4.7%	40.5	2.5%
UKBB GWAS	$P < 5e-8$ & $r^2 < 0.01$	DBP	VCDR	295	3.6%	55.1	1.8%
UKBB GWAS	$P < 5e-8$ & $r^2 < 0.01$	DBP	RNFL	983	5.0%	40.9	2.5%
UKBB GWAS	$P < 5e-8$ & $r^2 < 0.01$	DBP	GCC	983	5.0%	40.9	2.5%
Combined ICBP and UKBB GWAS. Khawaja *et al.* IOP GWAS.	$P < 5e-8$ & $r^2 < 0.01$	SBP and IOP	RNFL	917	-	SBP: 19.3<br>IOP: 2.3	-

Combined ICBP and UKBB GWAS. Khawaja *et al.* IOP GWAS.	$P < 5e-8$ & $r^2 < 0.01$	SBP and IOP	GCC	917	-	SBP: 19.3<br>IOP: 2.3	-

The R^2 value quantifies the proportion of the variance in the exposure explained by the genetic instrument and the F-statistic quantifies the strength of the relationship between the genetic instrument and exposure.

SNP-wise R^2 = 2*Effect^2*EAF*(1-EAF)/[2*Effect^2*EAF*(1-EAF)+SE^2*2*N*EAF*(1-EAF)] where EAF = effect allele frequency, SE = standard error and N = sample size.

Overall R^2 = sum of SNP-wise R^2 assuming independence of SNPs

F-stat = $((n-k-1)/k)*(R^2/(1-R^2))$.

Evangelou *et al.* 2018 = Combined ICBP and UKBB GWAS of SBP and DBP

Elsworth et. al. 2018 = UKBB GWAS of SBP and DBP

Table S3 - Mendelian randomization estimates for the effect of genetically predicted Systolic Blood Pressure (SBP) (Evangelou *et al.'s* combined ICBP & UKBB GWAS) on intraocular pressure (IOP) and primary open-angle glaucoma (POAG)

P-value and LD clumping threshold	Outcome	No. of SNPs	MR Method	Beta/OR (95% CI)	P-value	MR-Egger intercept P-value	MR PRESSO GlobHet P-value	No. of outlier SNPs detected from MR-PRESSO
$P < 5e\text{-}8$ & $r^2 < 0.01$	IOP	918	IVW	0.17 (0.11 - 0.24)	5.18E-07			
			Weighted Median	0.16 (0.09 - 0.23)	1.85E-05			
			ConMix	0.15 (0.09 - 0.24)	1.27E-04			
			MR-Egger	0.26 (0.04 - 0.48)	0.015	0.38		
		902	MR-PRESSO	0.20 (0.14 - 0.26)	1.37E-10		<0.001	16
$P < 1e\text{-}11$ & $r^2 < 0.01$	IOP	419	IVW	0.16 (0.07 – 0.24)	0.003			
			Weighted Median	0.17 (0.08 - 0.26)	0.002			
			ConMix	0.22 (0.06 - 0.33)	0.002			
			MR-Egger	0.26 (-0.03 – 0.56)	0.085			
		408	MR-PRESSO	0.17 (0.09 – 0.24)	1.27E-05		<0.001	11
$P < 5e\text{-}8$ & $r^2 < 0.01$	POAG	926	IVW	1.00 (0.94 - 1.06)	0.951			
			Weighted Median	0.99 (0.03 - 1.07)	0.870			
			ConMix	0.99 (0.92 - 1.08)	0.811			
			MR-Egger	1.11 (0.92 - 1.35)	0.267	0.234		
		917	MR-PRESSO	1.01 (0.95 - 1.07)	0.840		<0.001	9

MR effect estimates are scaled to a 10mmHg increase in SBP. IVW Beta (95% CI) is reported for IOP, and Odds Ratio (OR) (95% CI) is reported for POAG. IVW = inverse-variance weighted. CI = Confidence Interval. ConMix = Contamination Mixture. ICBP = International Consortium of Blood Pressure. UKBB = UK Biobank

Table S4 - Mendelian randomization estimates for the effect of genetically predicted Diastolic Blood Pressure (DBP) (Evangelou *et al.*'s combined ICBP & UKBB GWAS) on intraocular pressure (IOP) and primary open-angle glaucoma (POAG)

P-value and LD clumping threshold	Outcome	No. of SNPs	MR Method	Beta/OR (95% CI)	P-value	MR-Egger intercept P-value	MR PRESSO GlobHet P-value	No. of outlier SNPs detected from MR-PRESSO
$P < 5e-8$ & $r^2 < 0.01$	IOP	971	IVW	0.17 (0.05 - 0.28)	0.004			
			Weighted Median	0.14 (0.02 - 0.27)	0.025			
			ConMix	0.17 (0.01 - 0.32)	0.043			
			MR-Egger	0.16 (-0.2 - 0.52)	0.372	0.995		
		956	MR-PRESSO	0.16 (0.07 - 0.26)	3.59E-03		<0.001	15
$P < 1e-11$ & $r^2 < 0.01$	IOP	433	IVW	0.14 (0.00 – 0.29)	0.057			
			Weighted Median	0.14 (0.00 – 0.28)	0.056			
			ConMix	0.12 (-0.03 – 0.24)	0.143			
			MR-Egger	0.48 (-0.02 – 0.98)	0.060	0.166		
			MR-PRESSO	0.18 (0.08 – 0.29)	0.005		<0.001	12
$P < 5e-8$ & $r^2 < 0.01$	POAG	984	IVW	0.97 (0.88 - 1.07)	0.591			
			Weighted Median	0.93 (0.83 - 1.05)	0.230			
			ConMix	1.02 (0.87 - 1.17)	0.872			
			MR-Egger	1.05 (0.77 - 1.44)	0.740	0.599		
		979	MR-PRESSO	1.03 (0.94 - 1.14)	0.510		<0.001	5

MR effect estimates are scaled to a 10mmHg increase in DBP. IVW Beta (95% CI) is reported for IOP, and Odds Ratio (OR) (95% CI) is reported for POAG. IVW = inverse-variance weighted. CI = Confidence Interval. ConMix = Contamination Mixture. ICBP = International Consortium of Blood Pressure. UKBB = UK Biobank.

Table S5 - Mendelian randomization estimates for the effect of genetically predicted SBP and DBP (Evangelou *et al.'s* combined ICBP & UKBB GWAS) on macular retinal nerve fiber layer (mRNFL) thickness

P-value & LD clumping threshold	Exposure	Outcome	No. of SNPs	MR Method	Beta	P-value	MR-Egger intercept P-value	MR PRESSO GlobHet P-value	No. of outlier SNPs detected from MR-PRESSO
$P < 5e\text{-}8$ & $r^2 < 0.01$	SBP	mRNFL	926	IVW	-0.04 (-0.07 to -0.01)	0.004			
				Weighted Median	-0.04 (-0.08 to -0.003)	0.042			
				ConMix	-0.10 (-0.13 to -0.05)	0.0003			
				MR-Egger	-0.01 (-0.10 to 0.09)	0.871	0.424		
			922	MR-PRESSO	-0.04 (-0.07 to -0.01)	0.007		<0.001	4
$P < 1e\text{-}11$ & $r^2 < 0.01$	SBP	mRNFL	424	IVW	-0.04 (-0.08 to -0.004)	0.027			
				Weighted Median	-0.05 (-0.08 to -0.02)	0.022			
				ConMix	-0.12 (-0.17 to -0.07)	0.001			
				MR-Egger	-0.003 (-0.13 to 0.13)	0.954	0.539		
			420	MR-PRESSO	-0.04 (-0.08 to -0.01)	0.019		<0.001	4
$P < 5e\text{-}8$ & $r^2 < 0.01$	DBP	mRNFL	926	IVW	-0.03 (-0.08 to 0.02)	0.257			
				Weighted Median	-0.05 (-0.12 to 0.01)	0.127			
				ConMix	-0.15 (-0.22 to -0.06)	0.002			
				MR-Egger	0.02 (-0.14 to 0.18)	0.832	0.546		
			925	MR-PRESSO	-0.03 (-08 to 0.02)	0.307		<0.001	1

MR effect estimates are scaled to a 10mmHg increase in SBP. IVW Beta (95% CI) is reported for mRNFL. IVW = inverse-variance weighted. CI = Confidence Interval. ConMix = Contamination Mixture. ICBP = International Consortium of Blood Pressure. UKBB = UK Biobank

Table S6 - Mendelian randomization estimates for the effect of genetically predicted SBP and DBP (Evangelou *et al.'s* combined ICBP & UKBB GWAS) on ganglion cell complex (GCC) thickness

P-value and LD clumping threshold	Exposure	Outcome	No. of SNPs	MR Method	Beta	P-value	MR-Egger intercept P-value	MR PRESSO GlobHet P-value	No. of outlier SNPs detected from MR-PRESSO
$P < 5e\text{-}8$ & $r^2 < 0.01$	SBP	GCC	926	IVW	-0.04 (-0.07 to -0.01)	0.018			
				Weighted Median	-0.03 (-0.07 to 0.00)	0.082			
				ConMix	-0.06 (-0.11 to -0.01)	0.029			
				MR-Egger	-0.01 (-0.11 to 0.08)	0.801	0.589		
			920	MR-PRESSO	-0.03 (-0.06 to -0.004)	0.027		<0.001	6
$P < 1e\text{-}11$ & $r^2 < 0.01$	SBP	GCC	424	IVW	-0.04 (-0.08 to 0.00)	0.031			
				Weighted Median	-0.05 (-0.10 to 0.00)	0.033			
				ConMix	-0.02 (-0.07 to 0.02)	0.432			
				MR-Egger	0.01 (-0.13 to 0.14)	0.940	0.462		
			422	MR-PRESSO	-0.04 (-0.07 to -0.01)	0.023		<0.001	4
$P < 5e\text{-}8$ & $r^2 < 0.01$	DBP	GCC	983	IVW	-0.04 (-0.10 to 0.01)	0.121			
				Weighted Median	-0.04 (-0.10 to 0.03)	0.250			
				ConMix	-0.10 (-0.20 to -0.01)	0.043			
				MR-Egger	0.00 (-0.15 to 0.16)	0.969	0.574		
			981	MR-PRESSO	-0.04 (-0.09 to 0.01)	0.112		<0.001	2

MR effect estimates are scaled to a 10mmHg increase in SBP. IVW Beta (95% CI) is reported for GCC. IVW = inverse-variance weighted. CI = Confidence Interval. ConMix = Contamination Mixture. ICBP = International Consortium of Blood Pressure. UKBB = UK Biobank

Table S7 - Mendelian randomization estimates for the effect of genetically predicted Systolic Blood Pressure (SBP) (Elsworth *et al.'s* UKBB 2018 GWAS) on intraocular pressure (IOP) and primary open-angle glaucoma (POAG)

P-value and LD clumping threshold	Outcome	No. of SNPs	MR Method	Beta/OR (95% CI)	P-value	MR-Egger intercept P-value
P < 5e-8 & r^2 < 0.01	IOP	316	IVW	0.27 (0.09 - 0.44)	0.004	
			Weighted Median	0.22 (0.05 - 0.40)	0.011	
			ConMix	0.20 (-0.02 - 0.40)	0.078	
			MR-Egger	0.38 (-0.17 - 0.93)	0.173	0.659
P < 5e-8 & r^2 < 0.01	POAG	318	IVW	0.95 (0.82 - 1.09)	0.447	
			Weighted Median	1.00 (0.85 - 1.18)	0.978	
			ConMix	1.03 (0.89 - 1.20)	0.653	
			MR-Egger	0.91 (0.59 - 1.40)	0.658	0.835

MR effect estimates are scaled to a 1 standard deviation (SD) increase in SBP. IVW Beta (95% CI) is reported for IOP, and Odds Ratio (OR) (95% CI) is reported for POAG. IVW = inverse-variance weighted. CI = Confidence Interval. ConMix = Contamination Mixture. UKBB = UK Biobank.

Table S8 - Mendelian randomization estimates for the effect of genetically predicted Systolic Blood Pressure (SBP) and Diastolic Blood Pressure (DBP) (Elsworth *et al.'s* UKBB 2018 GWAS) on macular retinal nerve fiber layer thickness (mRNFL)

P-value and LD clumping threshold	Exposure	Outcome	No. of SNPs	MR Method	Beta/OR (95% CI)	P-value	MR-Egger intercept P-value
P < 5e-8 & $r^2 < 0.01$	SBP	mRNFL	319	IVW	-0.05 (-0.12 – 0.02)	0.146	
				Weighted Median	-0.08 (-0.17 - 0.01)	0.096	
				ConMix	-0.10 (-0.18 - -0.01)	0.025	
				MR-Egger	-0.05 (-0.27 – 0.17)	0.661	0.968
P < 5e-8 & $r^2 < 0.01$	DBP	mRNFL	308	IVW	-0.03 (-0.09 - 0.04)	0.460	
				Weighted Median	-0.05 (-0.13 – 0.04)	0.299	
				ConMix	-0.09 (-0.19 – 0.02)	0.105	
				MR-Egger	-0.11 (-0.30 – 0.10)	0.303	0.406

MR effect estimates are scaled to a 1 standard deviation (SD) increase in SBP. IVW Beta (95% CI) is reported for mRNFL. IVW = inverse-variance weighted. CI = Confidence Interval. ConMix = Contamination Mixture. UKBB = UK Biobank.

Table S9 - Mendelian randomization estimates for the effect of genetically predicted Systolic Blood Pressure (SBP) and Diastolic Blood Pressure (DBP) (Elsworth *et al.'s* UKBB 2018 GWAS) on ganglion cell complex (GCC) thickness

P-value and LD clumping threshold	Exposure	Outcome	No. of SNPs	MR Method	Beta/OR (95% CI)	P-value	MR-Egger intercept P-value
P < 5e-8 & $r^2 < 0.01$	SBP	GCC	319	IVW	-0.05 (-0.12 to 0.03)	0.228	
				Weighted Median	-0.05 (-0.15 to 0.37)	0.243	
				ConMix	-0.05 (-0.15 to 0.04)	0.252	
				MR-Egger	0.07 (-0.15 to 0.30)	0.531	0.280
P < 5e-8 & $r^2 < 0.01$	DBP	GCC	308	IVW	-0.03 (-0.10 to 0.04)	0.431	
				Weighted Median	-0.06 (-0.15 to 0.02)	0.156	
				ConMix	-0.14 (-0.23 to 0.06)	0.062	
				MR-Egger	-0.07 (-0.27 to 0.13)	0.494	0.655

MR effect estimates are scaled to a 1 standard deviation (SD) increase in SBP. IVW Beta (95% CI) is reported for GCC. IVW = inverse-variance weighted. CI = Confidence Interval. ConMix = Contamination Mixture. UKBB = UK Biobank.

Table S10 - Mendelian randomization estimates for the effect of genetically predicted Diastolic Blood Pressure (DBP) (Elsworth *et al.*'s UKBB 2018 GWAS) on intraocular pressure (IOP) and primary open-angle glaucoma (POAG)

P-value and LD clumping threshold	Outcome	No. of SNPs	MR Method	Beta/OR (95% CI)	P-value	MR-Egger intercept P-value	MR PRESSO GlobHet P-value	No. of outlier SNPs detected from MR-PRESSO
P < 5e-8 & r^2 < 0.01	IOP	307	IVW	0.21 (0.03 - 0.38)	0.020			
			Weighted Median	0.20 (0.03 - 0.36)	0.021			
			ConMix	0.36 (0.17 - 0.53)	3.47E-04			
			MR-Egger	0.31 (-0.22 - 0.84)	0.258	0.697		
P < 5e-8 & r^2 < 0.01	POAG	310	IVW	0.94 (0.80 - 1.10)	0.420			
			Weighted Median	1.04 (0.90 - 1.22)	0.568			
			ConMix	1.07 (0.94 - 1.23)	0.314			
			MR-Egger	1.26 (0.78 - 2.05)	0.344	0.201		

MR effect estimates are scaled to a 1 standard deviation (SD) increase in DBP. IVW Beta (95% CI) is reported for IOP, and Odds Ratio (OR) (95% CI) is reported for POAG. IVW = inverse-variance weighted. CI = Confidence Interval. ConMix = Contamination Mixture. UKBB = UK Biobank.

Table S11 - Mendelian randomization estimates for the effect of genetically predicted SBP and DBP (Evangelou *et al.'s* combined ICBP & UKBB GWAS) on vertical cup-to-disc ratio (VCDR) (Han *et al.* 2021)

P-value and LD clumping threshold	Exposure	Outcome	No. of SNPs	MR Method	Beta	P-value	MR-Egger intercept P-value	MR PRESSO GlobHet P-value	No. of outlier SNPs detected from MR-PRESSO
$P < 5e\text{-}8$ & $r^2 < 0.01$	SBP	VCDR	884	IVW	0.0004 (-0.002 – 0.003)	0.760			
				Weighted Median	-0.003 (-0.006 - -0.0003)	0.030			
				ConMix	-0.0004 (-0.003 - 0.002)	0.830			
				MR-Egger	0.005 (-0.003 – 0.013)	0.241	0.257		
			869	MR-PRESSO	0.0007 (-0.002 – 0.003)	0.538		<0.001	15
$P < 5e\text{-}8$ & $r^2 < 0.01$	DBP	VCDR	920	IVW	-0.001 (-0.004 – 0.002)	0.544			
				Weighted Median	0.002 (-0.002 – 0.006)	0.338			
				ConMix	-0.002 (-0.004 – 0.000)	0.304			
				MR-Egger	-0.003 (-0.010 – 0.004)	0.441	0.587		
			902	MR-PRESSO	0.001 (-0.003 – 0.005)	0.646		<0.001	18

MR effect estimates are scaled to a 10mmHg increase in SBP. IVW Beta (95% CI) is reported for VCDR. IVW = inverse-variance weighted. CI = Confidence Interval. ConMix = Contamination Mixture. ICBP = International Consortium of Blood Pressure. UKBB = UK Biobank

Table S12 - Mendelian randomization estimates for the effect of genetically predicted SBP and DBP (Elsworth *et al.'s* UKBB 2018 GWAS) on vertical cup-to-disc ratio (VCDR) (Han *et al.* 2021)

P-value and LD clumping threshold	Exposure	Outcome	No. of SNPs	MR Method	Beta	P-value	MR-Egger intercept P-value
$P < 5e-8$ & $r^2 < 0.01$	SBP	VCDR	297	IVW	-0.04 (-0.11 – 0.04)	0.330	
				Weighted Median	-0.04 (-0.11 – 0.03)	0.239	
				ConMix	0.02 (-0.05 – 0.06)	0.839	
				MR-Egger	-0.06 (-0.29 – 0.17)	0.608	0.835
$P < 5e-8$ & $r^2 < 0.01$	DBP	VCDR	295	IVW	-0.003 (-0.08 - 0.07)	0.947	
				Weighted Median	0.04 (-0.03 – 0.11)	0.305	
				ConMix	0.02 (-0.03 – 0.09)	0.300	
				MR-Egger	0.02 (-0.21 – 0.24)	0.879	0.853

MR effect estimates are scaled to a 1 standard deviation (SD) increase in SBP. IVW Beta (95% CI) is reported for VCDR. IVW = inverse-variance weighted. CI = Confidence Interval. ConMix = Contamination Mixture. UKBB = UK Biobank.

Table S13 - Mendelian randomization estimates for the effect of genetically predicted SBP and DBP (Evangelou *et al.'s* combined ICBP & UKBB GWAS) on vertical cup-to-disc ratio (VCDR) (Springelkamp *et al.* 2017)

P-value and LD clumping threshold	Exposure	Outcome	No. of SNPs	MR Method	Beta	P-value	MR-Egger intercept P-value	MR PRESSO GlobHet P-value	No. of outlier SNPs detected from MR-PRESSO
P < 5e-8 & r^2 < 0.01	SBP	VCDR	927	IVW	-0.003 (-0.008 – 0.002)	0.200			
				Weighted Median	-0.0005 (-0.008 – 0.007)	0.899			
				ConMix	-0.005 (-0.016 – 0.004)	0.265			
				MR-Egger	0.014 (-0.004 – 0.032)	0.132	0.048		
P < 5e-8 & r^2 < 0.01	DBP	VCDR	981	IVW	0.0008 (-0.008 - 0.010)	0.861			
				Weighted Median	-0.005 (-0.017 – 0.008)	0.446			
				ConMix	0.017 (0.007 – 0.027)	0.080			
				MR-Egger	-0.012 (-0.043 - 0.019)	0.461	0.410		

MR effect estimates are scaled to a 10mmHg increase in SBP. IVW Beta (95% CI) is reported for VCDR. IVW = inverse-variance weighted. CI = Confidence Interval. ConMix = Contamination Mixture. ICBP = International Consortium of Blood Pressure. UKBB = UK Biobank

Table S14 - Mendelian randomization estimates for the effect of genetically predicted SBP and DBP (Elsworth *et al.'s* UKBB 2018 GWAS) on vertical cup-to-disc ratio (VCDR) (Springelkamp *et al.* 2017)

**P-value and LD clumping threshold**	**Exposure**	**Outcome**	**No. of SNPs**	**MR Method**	**Beta**	**P-value**	**MR-Egger intercept P-value**
P < 5e-8 & r^2 < 0.01	SBP	VCDR	318	IVW	-8.23 x 10^{-3} (-0.14 – 0.12)	0.900	
				Weighted Median	0.047 (-0.11 – 0.21)	0.564	
				ConMix	0.092 (-0.065 – 0.27)	0.209	
				MR-Egger	-0.15 (-0.56 – 0.26)	0.469	0.471
P < 5e-8 & r^2 < 0.01	DBP	VCDR	311	IVW	-0.043 (-0.17 – 0.085)	0.511	
				Weighted Median	0.00 (-0.16 – 0.16)	1.00	
				ConMix	0.08 (-0.25 – 0.21)	0.549	
				MR-Egger	-0.28 (-0.68 – 0.12)	0.173	0.223

MR effect estimates are scaled to a 1 standard deviation (SD) increase in VCDR. IVW Beta (95% CI) is reported for GCC. IVW = inverse-variance weighted. CI = Confidence Interval. ConMix = Contamination Mixture. UKBB = UK Biobank.

Figure S1 – Scatter plot of the genetic associations of instrumental variants with systolic blood pressure and intraocular pressure.

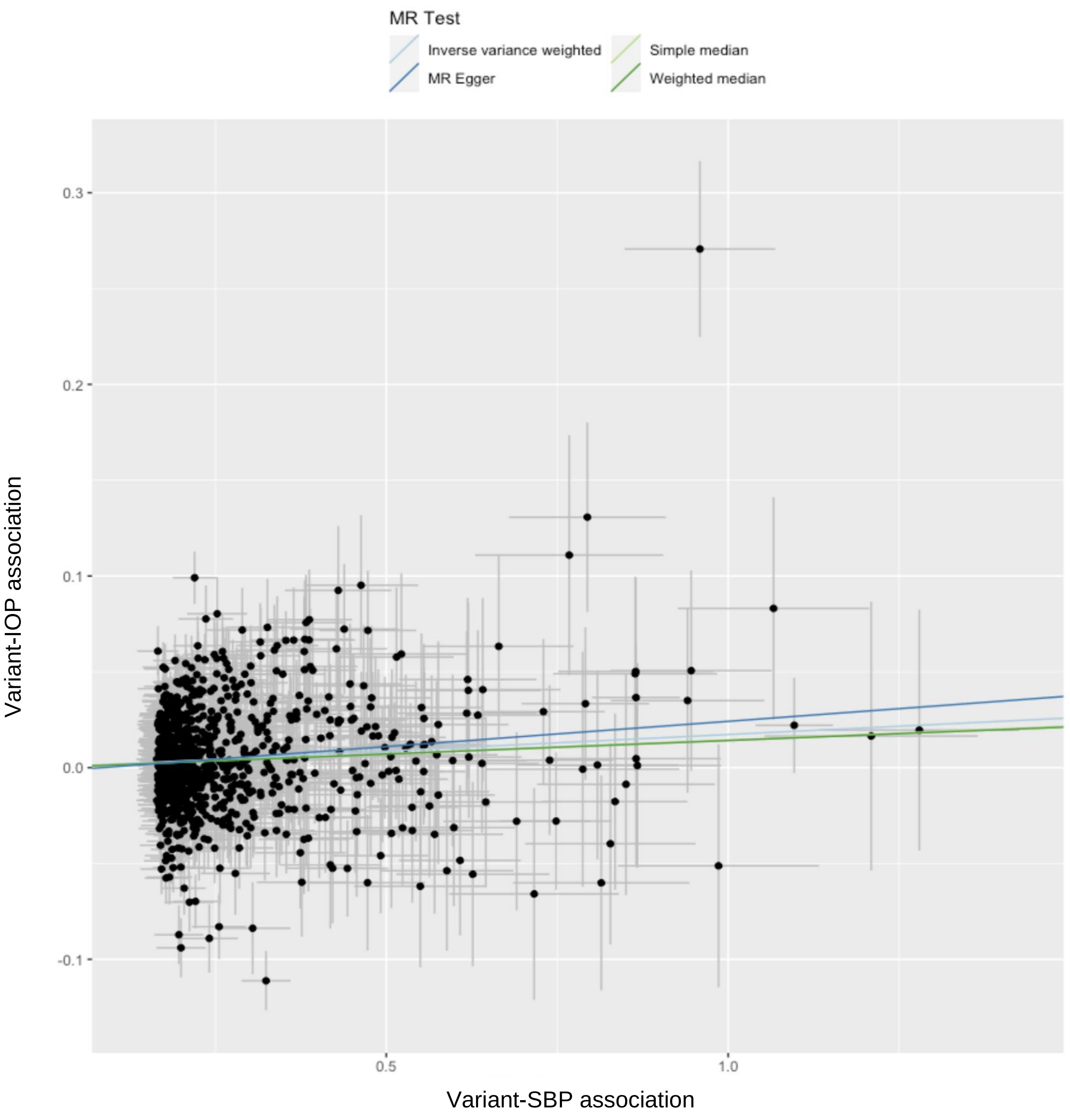

Figure S2 – Scatter plot of the genetic associations of instrumental variants with diastolic blood pressure and intraocular pressure.

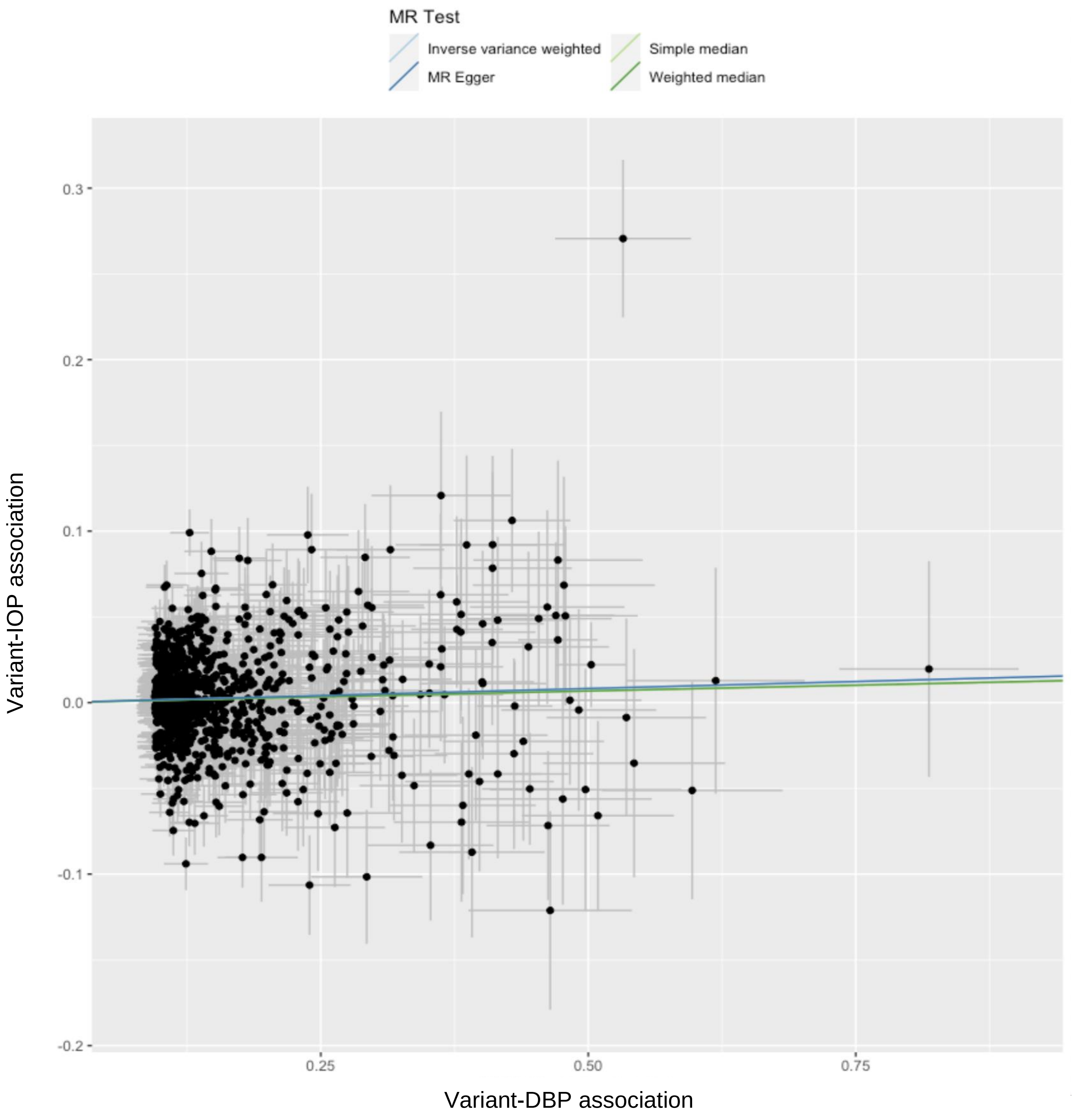

Figure S3 – Scatter plot of the genetic associations of instrumental variants with systolic blood pressure and mRNFL thickness.

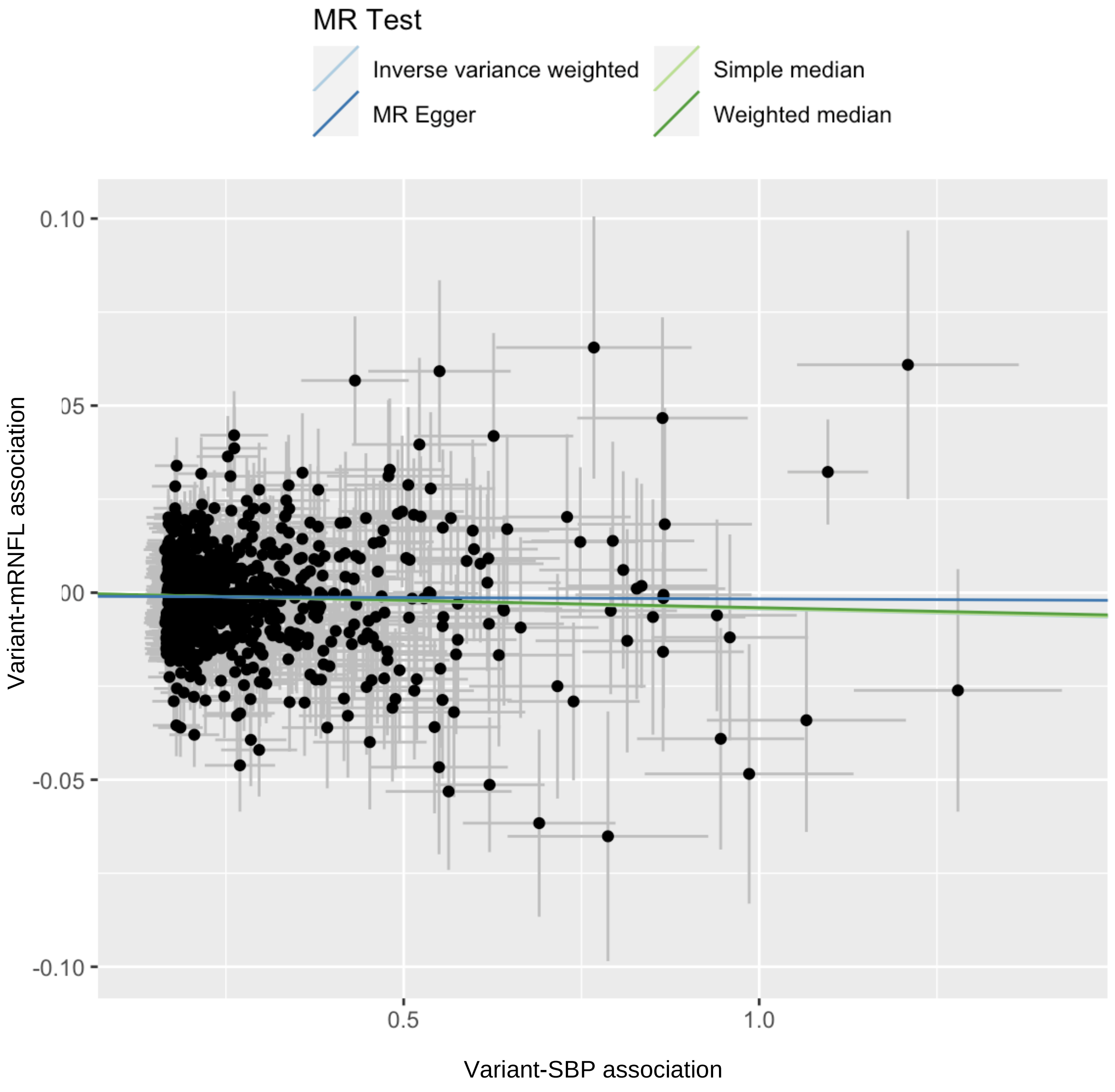

Figure S4 – Scatter plot of the genetic associations of instrumental variants with diastolic blood pressure and mRNFL thickness.

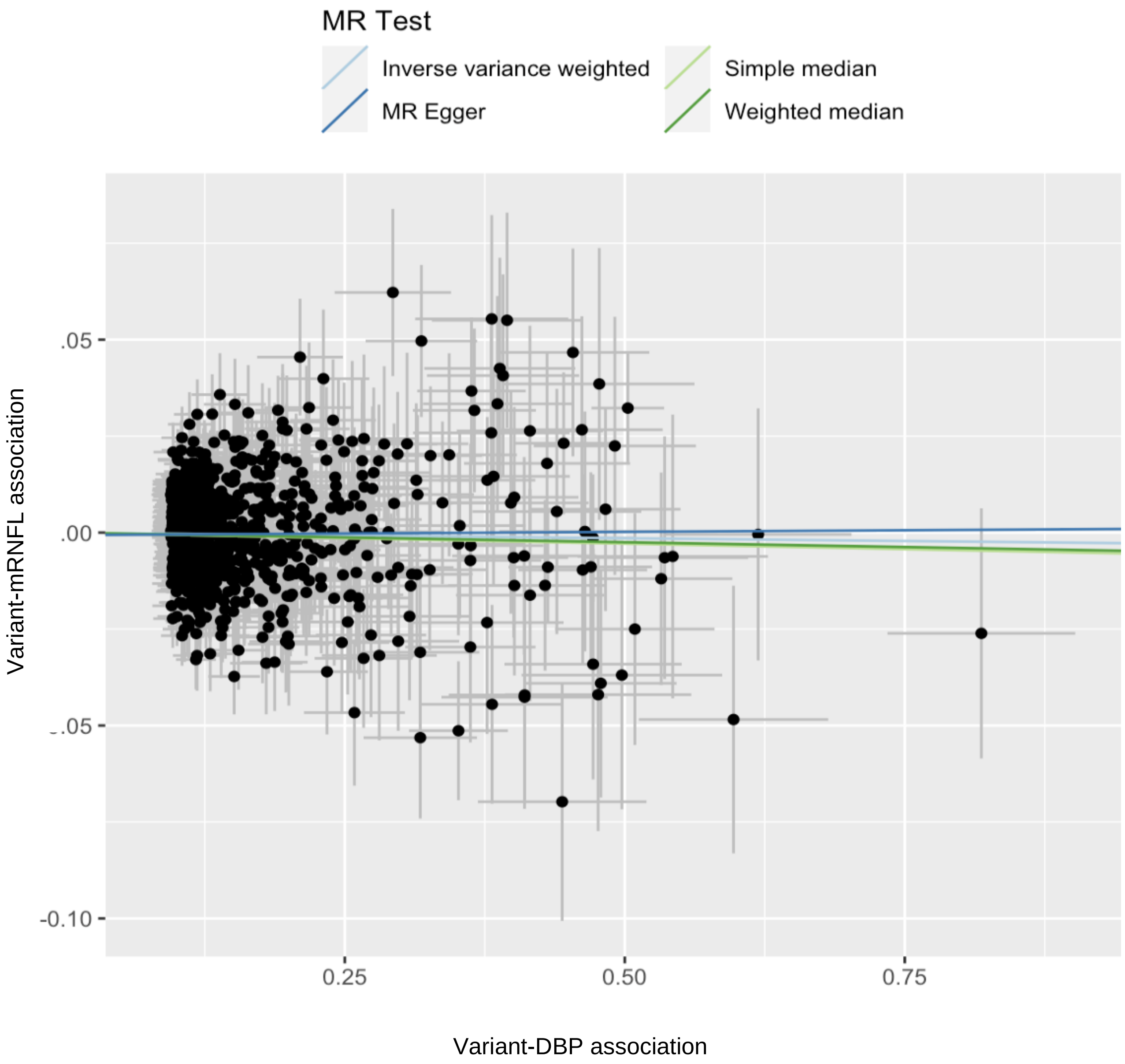

Figure S5 – Scatter plot of the genetic associations of instrumental variants with systolic blood pressure and GCC thickness.

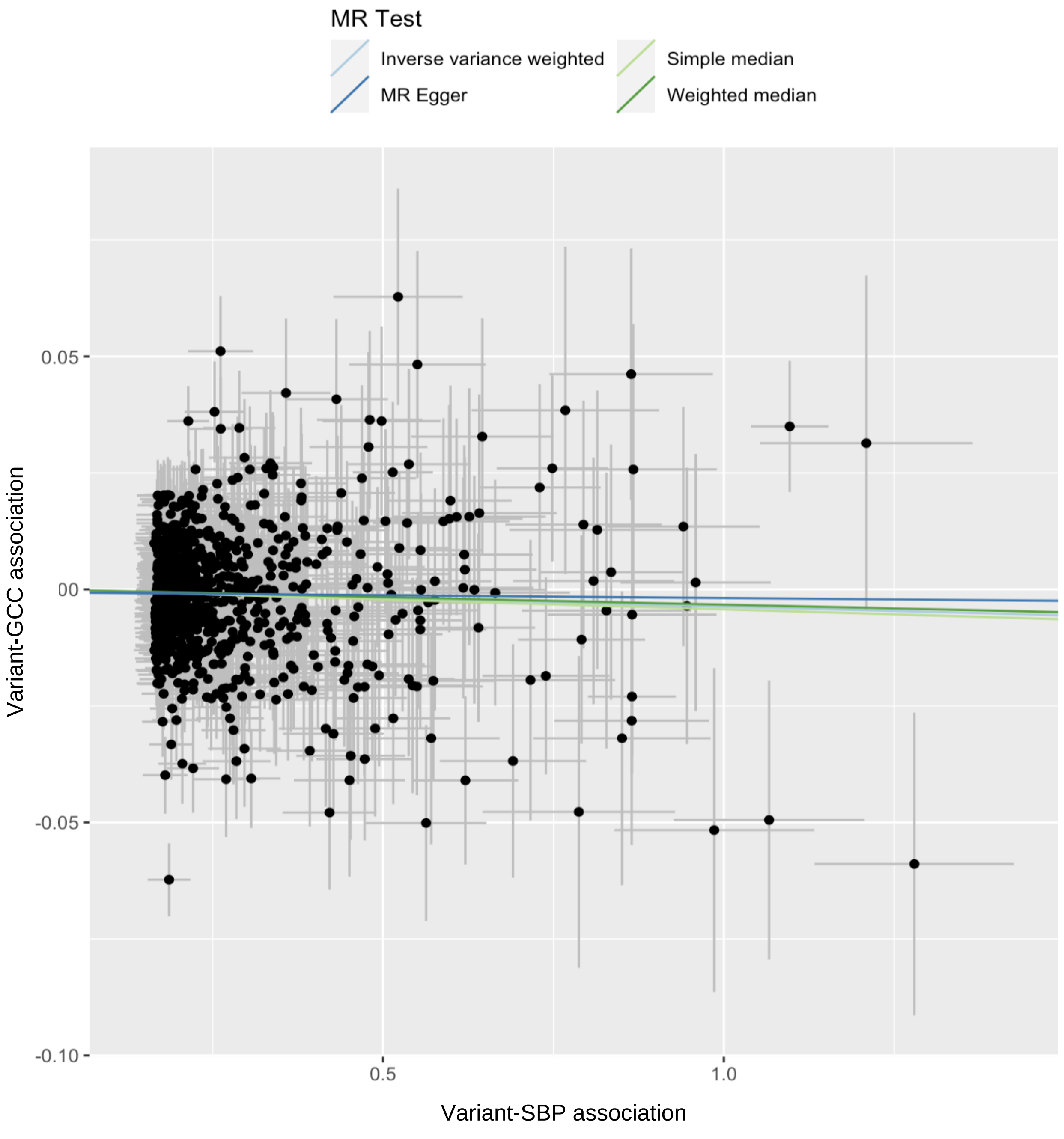

Figure S6 – Scatter plot of the genetic associations of instrumental variants with diastolic blood pressure and GCC thickness.

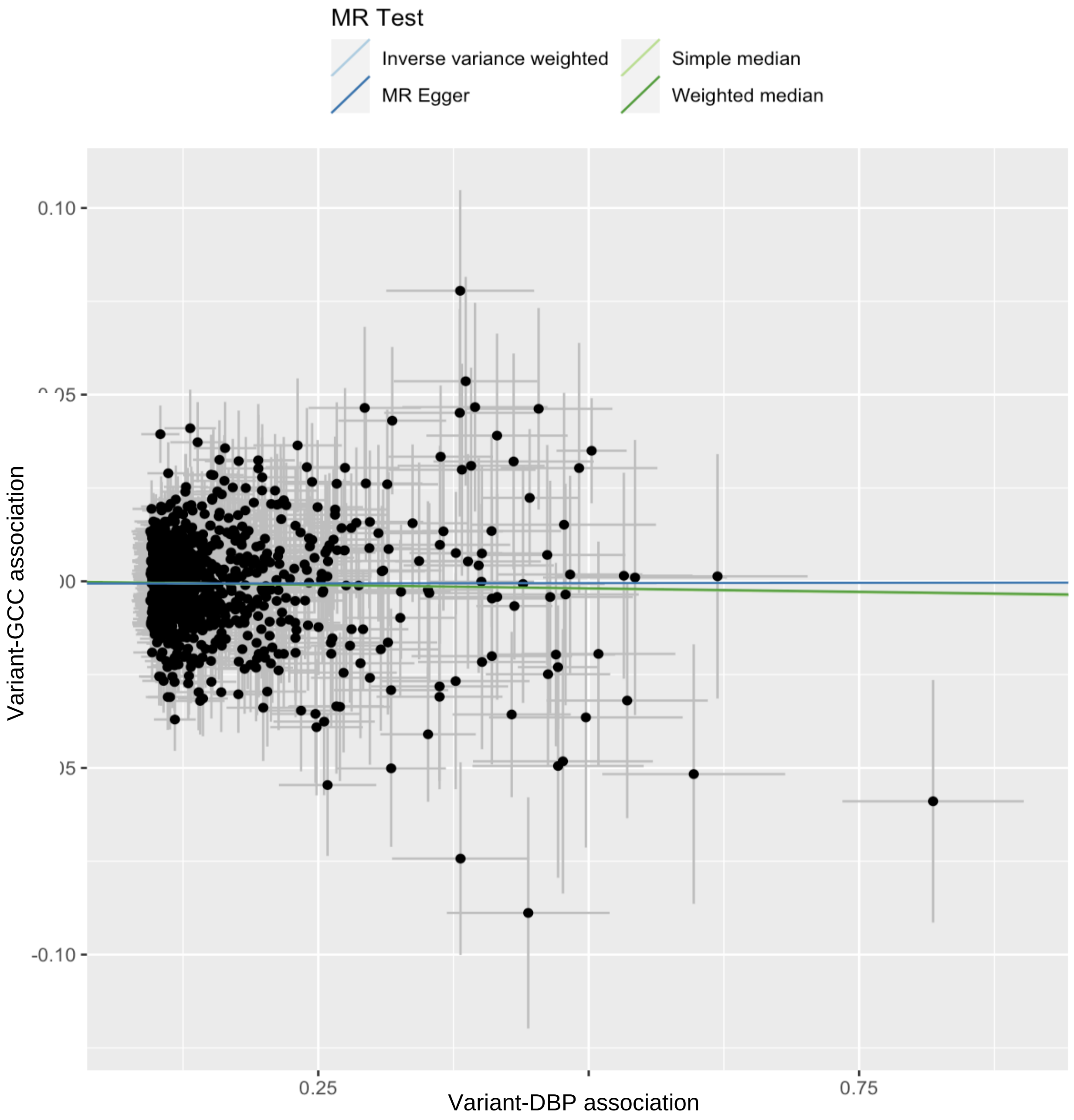

Figure S7 – Scatter plot of the genetic associations of instrumental variants with systolic blood pressure and primary open-angle glaucoma

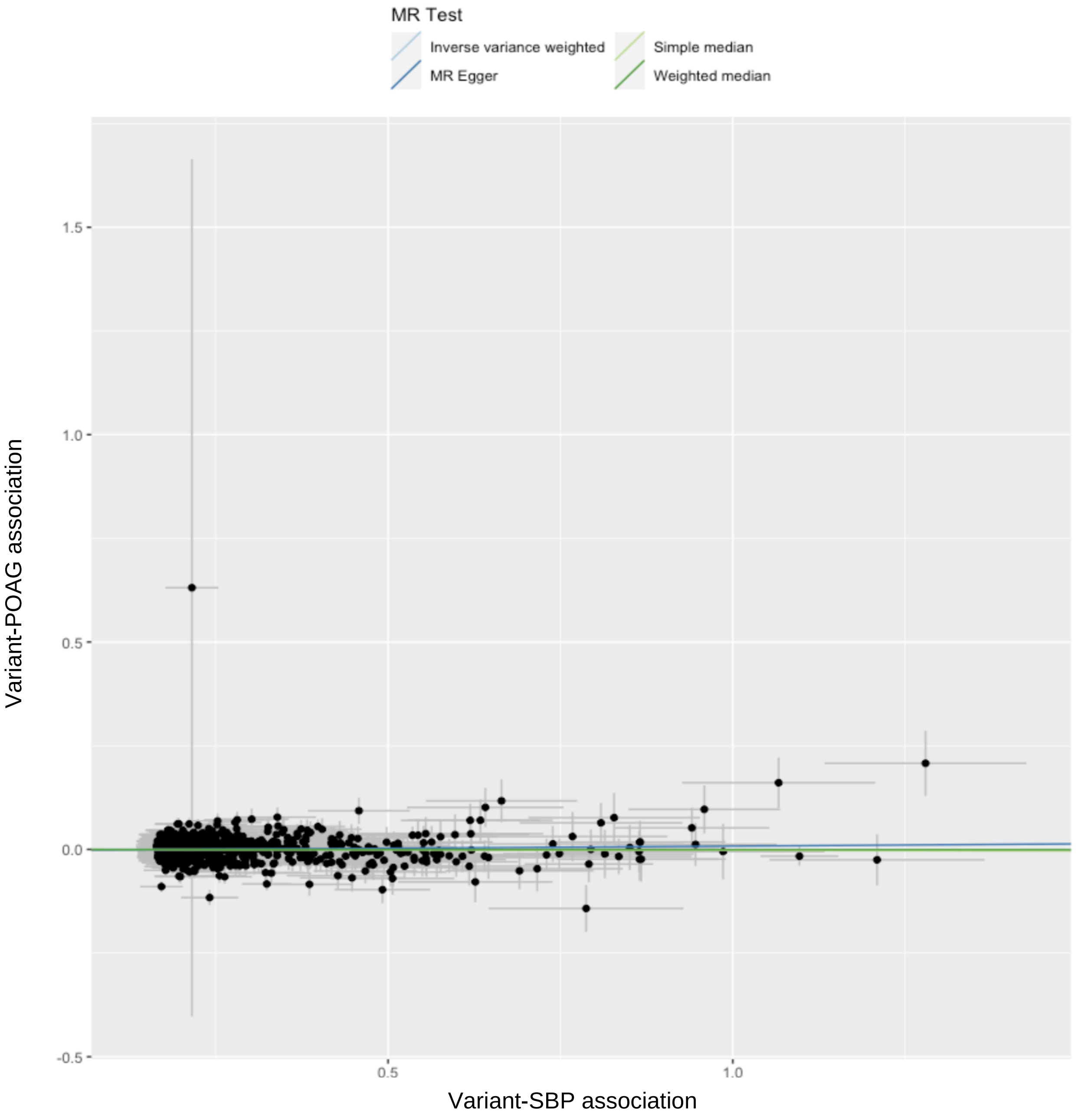

Figure S8 – Scatter plot of the genetic associations of instrumental variants with diastolic blood pressure and primary open-angle glaucoma

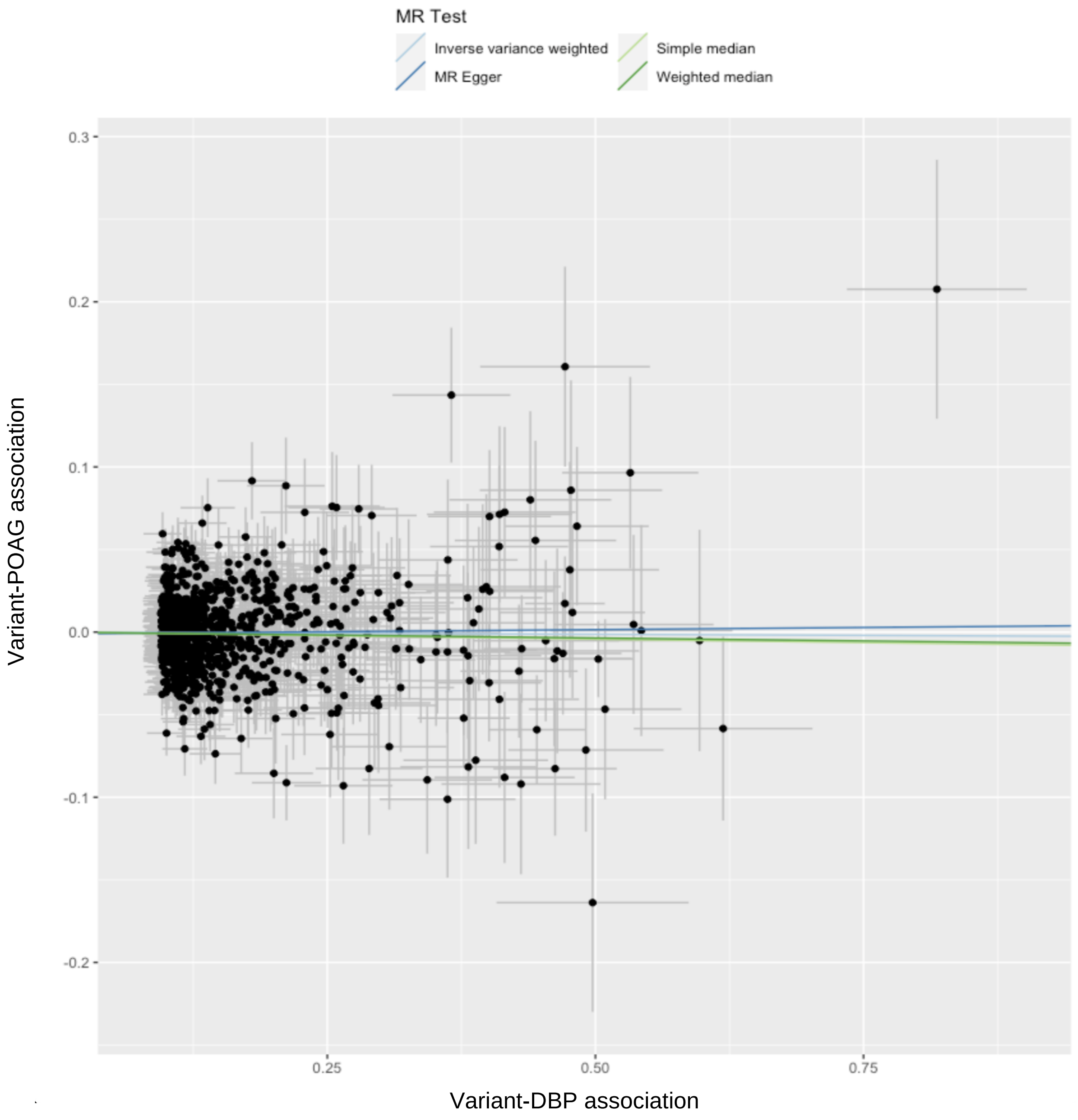

Figure S9 – Forest plot of MR estimates for blood pressure and VCDR

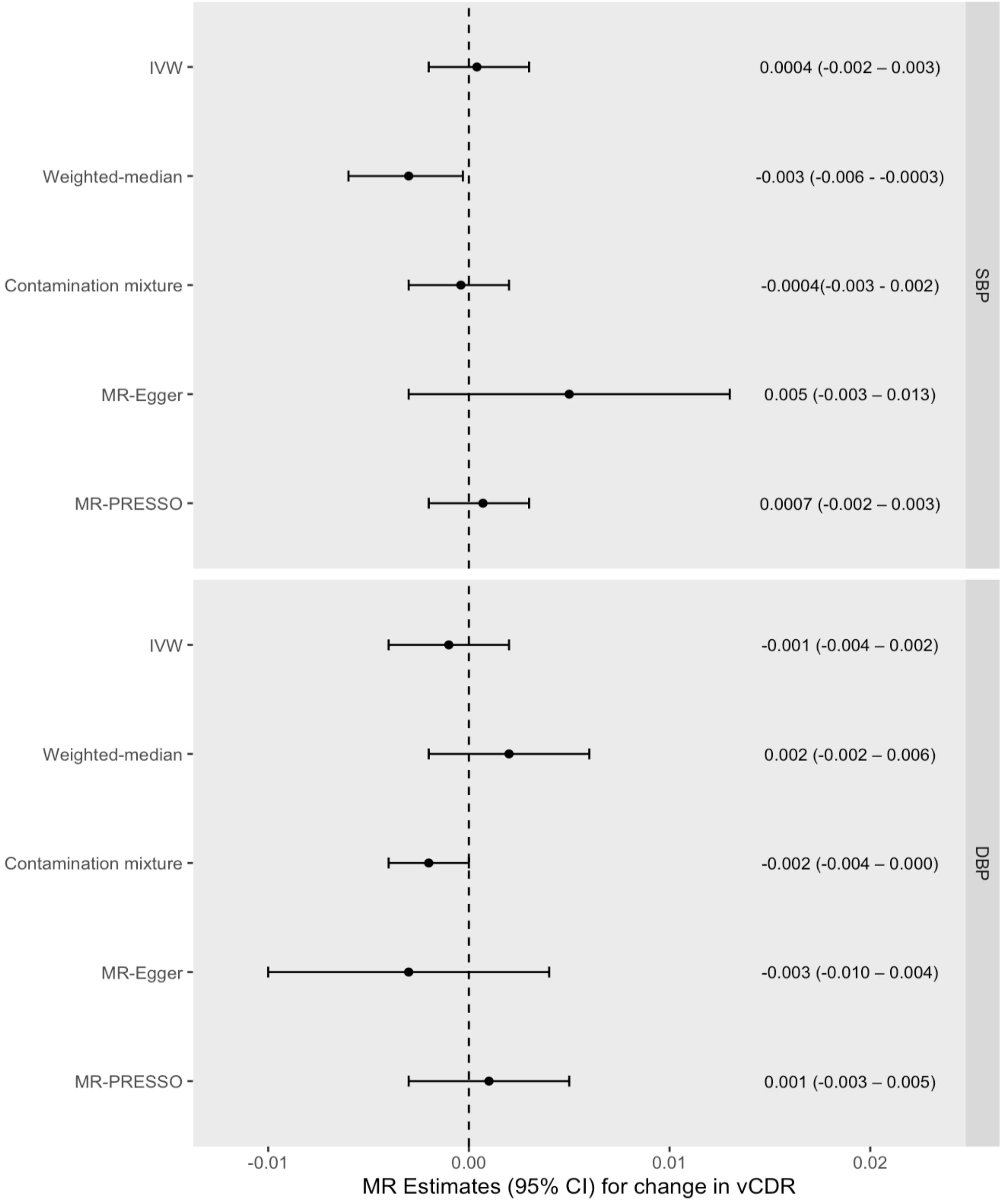

Figure S9 - MR effect estimates (beta coefficients) for the change in VCDR per 10mmHg increase in Systolic Blood Pressure (SBP) or Diastolic Blood Pressure (DBP). Primary estimate is the IVW = inverse variance-weighted. Pleiotropy-robust methods include MR-Egger, MR-PRESSO (Pleiotropy RESidual Sum and Outlier), Contamination mixture and Weighted-median methods.

Figure S10 – Scatter plot of the genetic associations of instrumental variants with systolic blood pressure and VCDR

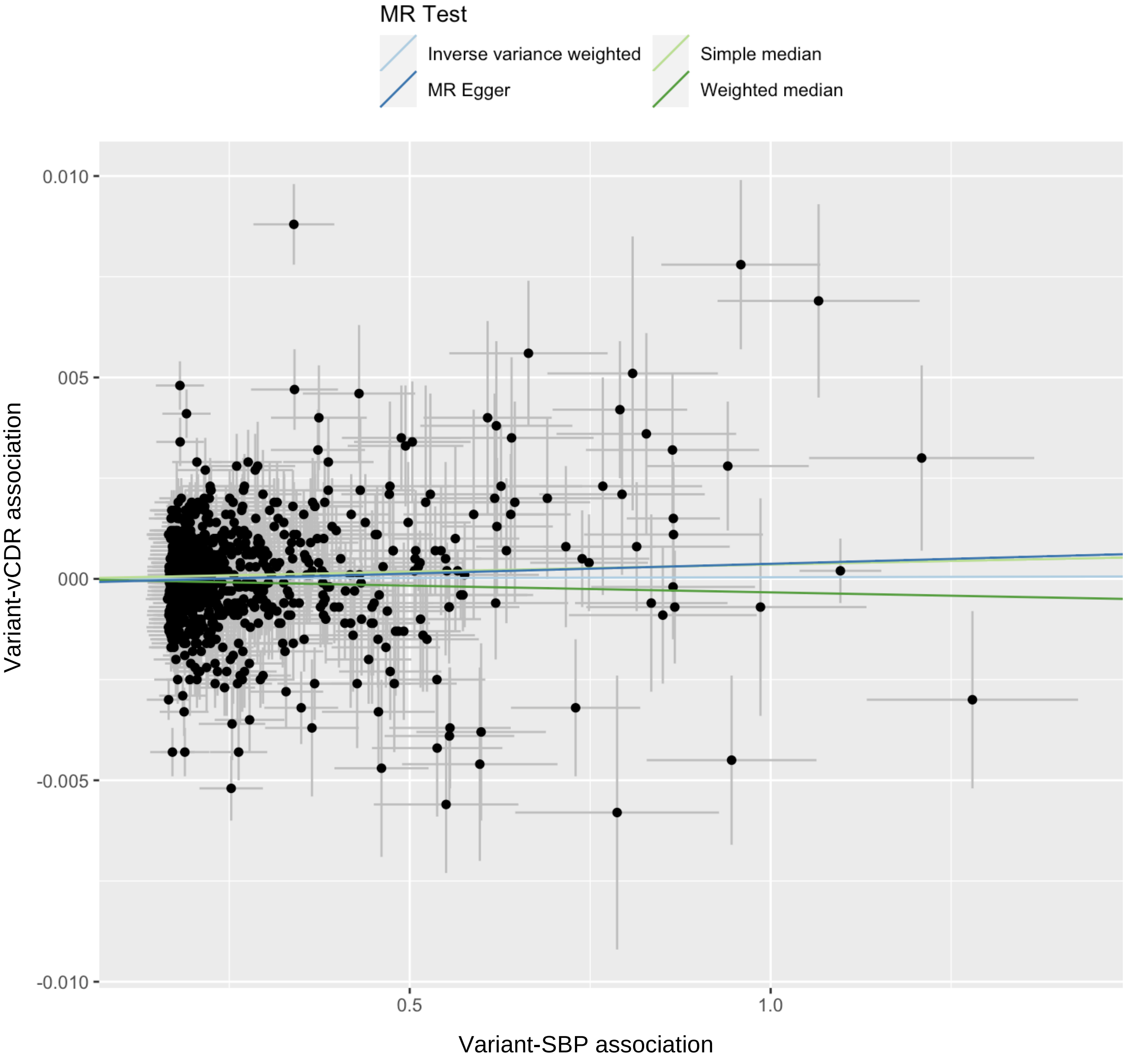

Figure S11 – Scatter plot of the genetic associations of instrumental variants with diastolic blood pressure and VCDR

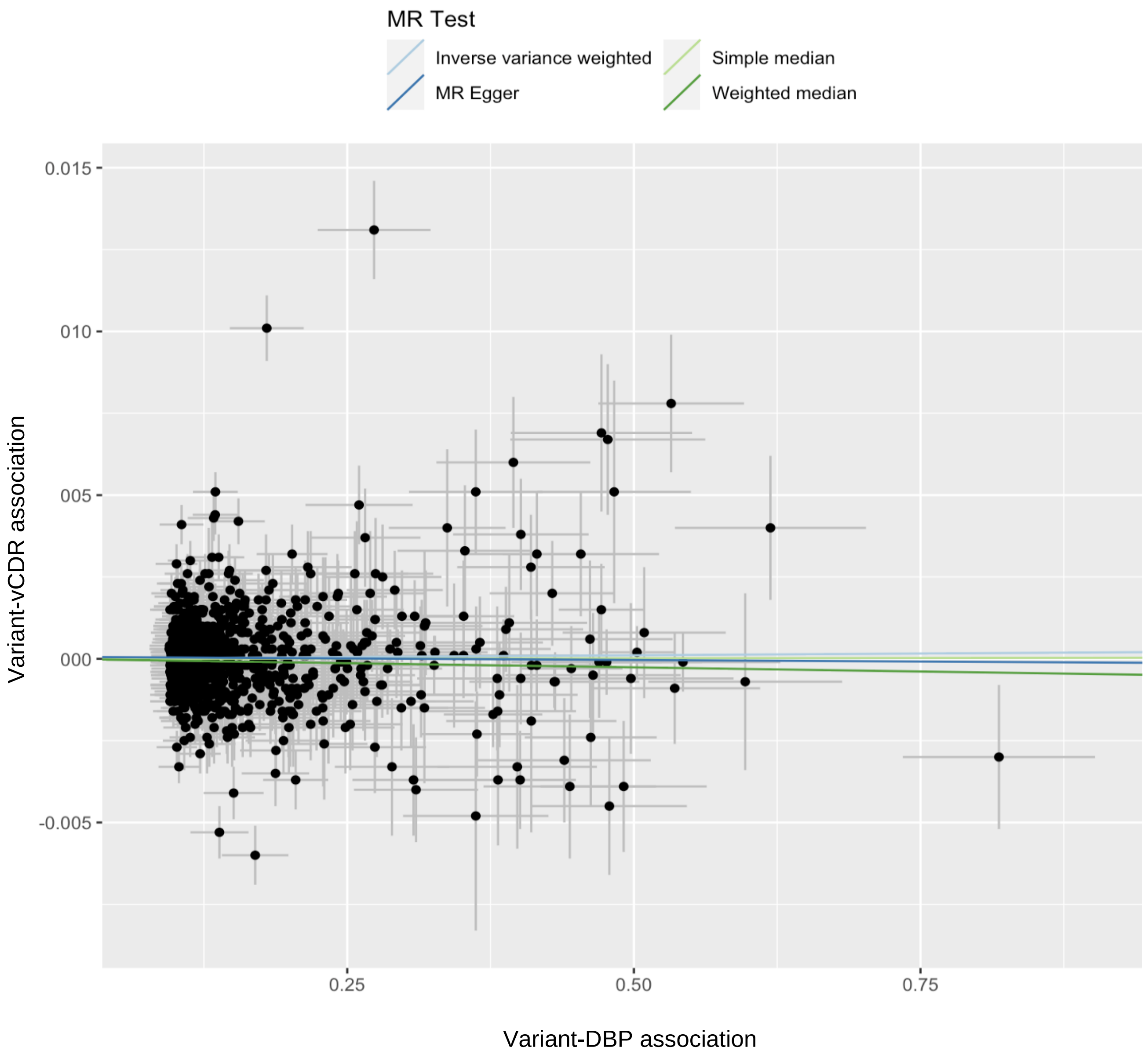

Table S15 - STROBE-MR Checklist

Item No.	*Section*	*Checklist item*	*Relevant text from manuscript*
1	**TITLE and ABSTRACT**	Indicate Mendelian randomization (MR) as the study's design in the title and/or the abstract if that is a main purpose of the study	See *Title* – Independent Effects of Blood Pressure on Intraocular Pressure and Retinal Ganglion Cell Degeneration: A Mendelian randomization study
	**INTRODUCTION**		
2	**Background**	Explain the scientific background and rationale for the reported study. What is the exposure? Is a potential causal relationship between exposure and outcome plausible? Justify why MR is a helpful method to address the study question	See *Introduction – explains that previous studies report positive associations of increased blood pressure with IOP, mRNFL thickness, GCC thickness, and POAG. However, there is vulnerability to unmeasured confounding in previous studies.*
3	**Objectives**	State specific objectives clearly, including pre-specified causal hypotheses (if any). State that MR is a method that, under specific assumptions, intends to estimate causal effects	See *Introduction - We therefore used MR to investigate the potential causal effect of systemic blood pressure on different glaucoma-related traits. First, we used univariable MR to investigate the causal effect of SBP and DBP on IOP, mRNFL thickness, GCC thickness, and liability to POAG. Then, for any significant associations identified between blood pressure and inner macular thinning, mediation analysis was conducted to investigate whether these effects were mediated by IOP.*<br><br>See *Methods – Univariable Mendelian randomization - Mendelian randomization leverages genetic variants as instruments within an instrumental variable (IV) framework and rests on three core assumptions.*
	**METHODS**		
4	**Study design and data sources**	Present key elements of the study design early in the article. Consider including a table listing sources of data for all phases of the study. For each data source contributing to the analysis, describe the following:	See *Methods* – Figure 1 and Table S1
	a)	Setting: Describe the study design and the underlying population, if possible. Describe the setting, locations, and relevant dates, including periods of recruitment, exposure, follow-up, and data collection, when available.	See Table S1.<br><br>Further details about the methodology of the original GWASs can be sought from the original publications, which are referenced accordingly.
	b)	Participants: Give the eligibility criteria, and the sources and methods of selection of participants. Report the sample size, and whether any power or sample size calculations were carried out prior to the main analysis	See Table S1<br><br>Given the use of prior GWAS data for exposures and outcomes, MR power calculations are not truly *a priori* and thus, were not carried out.

		c)	Describe measurement, quality control and selection of genetic variants	See *Methods – Data Sources* and *Genetic Instruments*
		d)	For each exposure, outcome, and other relevant variables, describe methods of assessment and diagnostic criteria for diseases	See Table S1.
		e)	Provide details of ethics committee approval and participant informed consent, if relevant	See *Methods – Data Sources* - Informed consent for all participants was obtained in the original studies, which were granted relevant ethical approval.
5	**Assumptions**		Explicitly state the three core IV assumptions for the main analysis (relevance, independence and exclusion restriction) as well as assumptions for any additional or sensitivity analysis	See *Methods – Univariable Mendelian randomization - First, the genetic instrument is robustly associated with the exposure. Second, the genetic instrument shares no common cause with the outcome. Third, the genetic instrument influences the outcome solely via the exposure.*<br><br>*Assumptions of pleiotropy-robust sensitivity analysis are explained in the Supplementary Methods.*
6	**Statistical methods: main analysis**		Describe statistical methods and statistics used	
	a)		Describe how quantitative variables were handled in the analyses (i.e., scale, units, model)	*See Methods – Univariable Mendelian randomization - MR estimates represent the change in IOP, mRNFL thickness, GCC thickness, VCDR, and odds ratio for POAG, per 10mmHg increase in SBP or DBP.*
	b)		Describe how genetic variants were handled in the analyses and, if applicable, how their weights were selected	*See Methods – Univariable Mendelian randomization - Genetic associations were harmonised by aligning effect alleles in both exposure and outcome datasets, with no exclusions made for palindromic variants. In the primary analysis, genetic variants were pooled via the inverse-variance weighted (IVW) approach. Weighted median, contamination mixture, MR-Egger and MR-PRESSO methods were also used.*
	c)		Describe the MR estimator (e.g. two-stage least squares, Wald ratio) and related statistics. Detail the included covariates and, in case of two-sample MR, whether the same covariate set was used for adjustment in the two samples	*See Methods – Univariable Mendelian randomization - MR estimates were generated by first calculating the Wald ratio for each variant, i.e., variant-outcome association divided by the variant-exposure association.*<br><br>*See Methods – Data Sources - genetic association data for SBP and DBP were derived from Evangelou et al.'s GWAS meta-analysis of UKBB and ICBP (N = 757,601). These GWASs were adjusted for BMI, and corrected for antihypertensive medication use by adding 15mmHg.*
	d)		Explain how missing data were addressed	*Not applicable (only summary data used).*
	e)		If applicable, indicate how multiple testing was addressed	*No correction was made for multiple testing*

7	**Assessment of assumptions**	Describe any methods or prior knowledge used to assess the assumptions or justify their validity	*IV1 - F-statistics were calculated to quantify the strength of the association between the instrument and the exposure. See Supplementary Table S2. IV3 - The IVW approach assumes no horizontal pleiotropy and so a series of sensitivity analyses, including weighted median, contamination mixture, MR-Egger and MR-PRESSO methods were used to interrogate the robustness of results to horizontal pleiotropy. Further details of these methods can be found in the Supplementary Methods.*
8	**Sensitivity analyses and additional analyses**	Describe any sensitivity analyses or additional analyses performed (e.g. comparison of effect estimates from different approaches, independent replication, bias analytic techniques, validation of instruments, simulations)	*See Methods - Sensitivity analyses for pleiotropy, winner's curse, and collider bias. See Tables S3-14 for the results of these sensitivity analyses.*
9	**Software and preregistration**		
	a)	Name statistical software and package(s), including version and settings used	*MR analyses were performed using the TwoSampleMR, MendelianRandomization, MR-PRESSO and MVMR packages in R (version 4.1.2).*
	b)	State whether the study protocol and details were pre-registered (as well as when and where)	*Study protocol was not pre-registered.*
	**RESULTS**		
10	**Descriptive data**		
	a)	Report the numbers of individuals at each stage of included studies and reasons for exclusion. Consider use of a flow diagram	*Figure 1 provides flow diagram of study design inc. number of participants. No participants were excluded.*
	b)	Report summary statistics for phenotypic exposure(s), outcome(s), and other relevant variables (e.g., means, SDs, proportions)	*See Table S1.*
	c)	If the data sources include meta-analyses of previous studies, provide the assessments of heterogeneity across these studies	*Full details of assessments of heterogeneity can be found in the original publications of the GWASs.* .
	d)	For two-sample MR:<br>i. Provide justification of the similarity of the genetic variant-exposure associations between the exposure and outcome samples<br>ii. Provide information on the number of individuals who	*i) All studies used almost exclusively European ancestry individuals.*<br>*ii) There is some overlap between the exposure dataset and the outcome dataset but one cannot ascertain from the precise numbers from the available information on these datasets. We calculate F-statistics (see Table S2) showing a low risk of weak instrument bias and use Qhet as an MVMR sensitivity analysis. Thus,*

		overlap between the exposure and outcome studies	*such sample overlap is unlikely to material affect our results.*
11	**Main results**		
	a)	Report the associations between genetic variant and exposure, and between genetic variant and outcome, preferably on an interpretable scale	*See Results, Figures 2-5 and Tables S3-14*
	b)	Report MR estimates of the relationship between exposure and outcome, and the measures of uncertainty from the MR analysis, on an interpretable scale, such as odds ratio or relative risk per SD difference	*See Results, Figures 2-5 and Tables S3-14*
	c)	If relevant, consider translating estimates of relative risk into absolute risk for a meaningful time period	*Not applicable*
	d)	Consider plots to visualize results (e.g. forest plot, scatterplot of associations between genetic variants and outcome versus between genetic variants and exposure)	*Forest plots are illustrated in Figures 2-5 in the main manuscript and Figure S9 in Supplement. Scatter plots illustrated in Figures S1-8, S10 and S11.*
12	**Assessment of assumptions**		
	a)	Report the assessment of the validity of the assumptions	*Validity of IV assumptions discussed Results and Discussion sections.*
	b)	Report any additional statistics (e.g., assessments of heterogeneity across genetic variants, such as I^2, Q statistic or E-value)	*Calculations of R^2 and F-statistics discussed in Methods – Genetic Instruments. R^2 and F-statistics are detailed in Table S2.*
13	**Sensitivity analyses and additional analyses**		
	a)	Report any sensitivity analyses to assess the robustness of the main results to violations of the assumptions	*See Results discussion of pleiotropy-robust sensitivity analyses, Figures 2-5 and Tables S3-14*
	b)	Report results from other sensitivity analyses or additional analyses	*See Results discussion of winner's curse and collider bias sensitivity analyses, Figures 2-5 and Tables S3-14*
	c)	Report any assessment of direction of causal relationship (e.g., bidirectional MR)	*Direction of MR estimates stated in Results and Discussion.*
	d)	When relevant, report and compare with estimates from non-MR analyses	*See Discussion for comparison to prior observational evidence.*

	e)	Consider additional plots to visualize results (e.g., leave-one-out analyses)	*Scatter plots illustrated in Figures S1-8, S10 and S11.*
	**DISCUSSION**		
14	**Key results**	Summarize key results with reference to study objectives	*See Discussion - Principal findings in context*
15	**Limitations**	Discuss limitations of the study, taking into account the validity of the IV assumptions, other sources of potential bias, and imprecision. Discuss both direction and magnitude of any potential bias and any efforts to address them	*See Discussion - Limitations*
16	**Interpretation**		
	a)	Meaning: Give a cautious overall interpretation of results in the context of their limitations and in comparison with other studies	*See Discussion, in particular, Principal findings in context and Conclusions.*
	b)	Mechanism: Discuss underlying biological mechanisms that could drive a potential causal relationship between the investigated exposure and the outcome, and whether the gene-environment equivalence assumption is reasonable. Use causal language carefully, clarifying that IV estimates may provide causal effects only under certain assumptions	*See Discussion - Blood pressure and intraocular pressure. A range of mechanisms have been hypothesized to explain the link between blood pressure and IOP. Elevated blood pressure may increase ciliary perfusion pressures and lead to greater ultrafiltration of aqueous fluid in the ciliary body. Increased blood pressure may also increase episcleral venous pressures and reduce aqueous humour outflow.*<br><br>*See Discussion – Blood pressure and retinal ganglion cell degeneration. POAG is known to arise across a spectrum of IOP, including within the normal range, and vascular dysfunction, e.g., endothelial dysfunction and impaired autoregulatory reserve, is one proposed category of IOP-independent mechanisms in the pathogenesis of POAG. Mechanistic studies will be important in exploring whether such vascular dysfunction mediates the observed effect of SBP on retinal ganglion cell degeneration or whether distinct biological mechanisms are involved.*
	c)	Clinical relevance: Discuss whether the results have clinical or public policy relevance, and to what extent they inform effect sizes of possible interventions	*See Discussion – Blood Pressure and Intraocular pressure. Clinically, it should be noted that increases in IOP do not necessarily lead to ocular hypertension and not every individual with ocular hypertension necessarily develops POAG. However, given prior evidence demonstrating that lowering IOP from any baseline level slows disease progression in patients with POAG, the effect of blood pressure on IOP may be of particular clinical relevance in those individuals with, or at high risk of developing, POAG.*

			See Conclusion – This implies that targeted blood pressure control, for instance through lifestyle modification and antihypertensive medication, could help preserve vision by lowering IOP and by preventing retinal ganglion cell degeneration, including in individuals with a normal eye pressure.
17	**Generalizability**	Discuss the generalizability of the study results (a) to other populations, (b) across other exposure periods/timings, and (c) across other levels of exposure	*See Discussion – Limitations - Finally, given that the frequency and distribution of genetic variants differ across ancestries, we restricted our analysis to European ancestry individuals to avoid confounding by ancestry. Consequently, these findings may not be generalisable to other ancestries.*
	**OTHER INFORMATION**		
18	**Funding**	Describe sources of funding and the role of funders in the present study and, if applicable, sources of funding for the databases and original study or studies on which the present study is based	*See Acknowledgements – Funding/ Support.*
19	**Data and data sharing**	Provide the data used to perform all analyses or report where and how the data can be accessed, and reference these sources in the article. Provide the statistical code needed to reproduce the results in the article, or report whether the code is publicly accessible and if so, where	*Exposure GWAS data and POAG outcome data are publicly available. Our IOP, mRNFL and GCC thickness outcome data are not currently publicly available.*<br><br>*Code for analyses conducted with TwoSampleMR and MendelianRandomization packages in R (version 4.1.2) are publicly available at https://github.com/MRCIEU/TwoSampleMR and https://github.com/cran/MendelianRandomization.*
20	**Conflicts of Interest**	All authors should declare all potential conflicts of interest	*See Acknowledgements - Conflicts of interest disclosures*

References

[1] Burgess S, Small DS, Thompson SG. A review of instrumental variable estimators for Mendelian randomization. *Stat Methods Med Res.* 2017;26(5):2333-2355.

[2] Burgess S, Butterworth A, Thompson SG. Mendelian randomization analysis with multiple genetic variants using summarized data. *Genet Epidemiol.* 2013;37(7):658-665.

[3] Bowden J, Davey Smith G, Haycock PC, Burgess S. Consistent Estimation in Mendelian Randomization with Some Invalid Instruments Using a Weighted Median Estimator. *Genet Epidemiol.* 2016;40(4):304-314.

[4] Burgess S, Foley CN, Allara E, Staley JR, Howson JMM. A robust and efficient method for Mendelian randomization with hundreds of genetic variants. *Nat Commun.* 2020;11(1):376.

[5] Bowden J, Davey Smith G, Burgess S. Mendelian randomization with invalid instruments: effect estimation and bias detection through Egger regression. *Int J Epidemiol.* 2015;44(2):512-525.

[6] Verbanck M, Chen CY, Neale B, Do R. Detection of widespread horizontal pleiotropy in causal relationships inferred from Mendelian randomization between complex traits and diseases. *Nat Genet.* 2018;50(5):693-698.
